# Disentangling Crowdfunding from Fraudfunding

**DOI:** 10.1007/s10551-021-04942-w

**Published:** 2021-10-20

**Authors:** Douglas Cumming, Lars Hornuf, Moein Karami, Denis Schweizer

**Affiliations:** 1 College of Business, Florida Atlantic University, 777 Glades Road, Boca Raton, FL 33431 USA; 2 Birmingham Business School, University of Birmingham, University House, 116 Edgbaston Park Rd, Birmingham, B15 2TY UK; 3 Faculty of Business Studies and Economics, University of Bremen, Max-von-Laue-Str. 1, 28334 Bremen, Germany; 4 John Molson School of Business, Concordia University, 1450 Rue Guy, Montreal, QC H3H 0A1 Canada; 5 CESifo Research Network, Poschingerstraße 5, 81679 Munich, Germany

**Keywords:** Crowdfunding, Entrepreneurial finance, Fraud, Internet

## Abstract

Fraud in the reward-based crowdfunding market has been of concern to regulators, but it is arguably of greater importance to the nascent industry itself. Despite its significance for entrepreneurial finance, our knowledge of the occurrence, determinants, and consequences of fraud in this market, as well as the implications for the business ethics literature, remain limited. In this study, we conduct an exhaustive search of all media reports on Kickstarter campaign fraud allegations from 2010 through 2015. We then follow up until 2018 to assess the ultimate outcome of each allegedly fraudulent campaign. First, we construct a sample of 193 fraud cases, and categorize them into *detected* vs. *suspected* fraud, based on a set of well-defined criteria. Next, using multiple matched samples of non-fraudulent campaigns, we determine which features are associated with a higher probability of fraudulent behavior. Second, we document the short-term negative consequences of possible breaches of trust in the market, using a sample of more than 270,000 crowdfunding campaigns from 2010 through 2018 on Kickstarter. Our results show that crowdfunding projects launched around the public announcement of a late and significant misconduct detection (resulting in suspension) tend to have a lower probability of success, raise less funds, and attract fewer backers.

**Supplementary Information:**

The online version contains supplementary material available at 10.1007/s10551-021-04942-w.

It’s a credit to Kickstarter and the collective power of the crowd to identify fraud….

-CNN Money, June 17, 2013^1^

If you utter the word “crowdfunding” in front of a dusty old-fashioned securities lawyer, make sure you have a fully charged defibrillator on hand. Perhaps a fully equipped contingent of ER doctors and nurses. It won’t be pretty.

-Financial Post, July 31, 2013^2^

## Introduction

Reward-based crowdfunding (hereafter, crowdfunding) has emerged in recent years as a catalyst for entrepreneurship, an important new means of financing early-stage ventures, and a door opener for successful financing. As an alternative solution to the capital gap problem for start-ups, crowdfunding can complement or substitute for other sources of financing, such as venture capital or angel investors. Early-stage ventures have benefited enormously from its availability, and its positive impact on new firm creation and future venture capital investments has become increasingly evident (Assenova et al., 2016; Sorenson et al., 2016). This highlights the importance of investigating any issues that could negatively affect the crowdfunding market and endanger its long-term existence.

Trust between counterparties in any economic exchange is vital (Brockman et al., 2020; Hain et al., 2016). Therefore, crowdfunding adoption depends significantly on establishing trust in the market. Equity markets have demonstrated the fragility of trust, and how a breach can not only negatively affect specific firms (Davidson & Worrel, 1988), but result in the collapse of entire market segments (Hainz, 2018). The concept of the *Trust Triangle* was recently adapted for financial markets and fraud (Dupont & Karpoff, 2019). According to this framework, firms can *ex ante* invest in accountability and build trust through three main channels: first-party, related-party, and third-party enforcement (the first, second, and third legs of the Trust Triangle). The three legs are not equally effective in a crowdfunding context. The crowdfunding market is still in its infancy, and campaign creators have no legal obligation, for example, to provide income statements or profit and loss accounts to the platform or regulatory bodies. This suggests somewhat weak third-party enforcement in the market. Backers must trust campaign creators to use the funds obtained to deliver on their promises (first-party enforcement), and trust the platform to conduct thorough pre-screening of projects before they are posted (related-party enforcement). Thus, one of the core elements of a functional crowdfunding market is trust among backers, campaign creators, and the platform.

Incidents of fraudulent behavior by campaign creators, and the inactivity of platforms to prevent them, can negatively affect the open-mindedness of crowdfunding backers. Therefore, it is important to document fraudulent cases to (1) assess which factors signal weak first-party enforcement and help predict subsequent fraud, and (2) identify incidents that lead to a breach of trust associated with weak related-party enforcement and analyze their consequences.

In the first part of our empirical analyses (*Determinants of Fraud*), we categorize fraudulent behavior based on Kickstarter campaign fraud allegation reports from 2010 to 2015. We follow these cases until 2018 to assess the outcomes. We conduct a methodical search of media reports, and use specific criteria to finalize a sample of campaigns associated with fraudulent behavior. Using this sample and multiple matched samples of non-fraudulent campaigns, we find that fraudsters are less likely to have engaged in prior crowdfunding activities and to use social media, such as Facebook. We also find that fraudsters tend to offer a higher number of enticements through pledge categories, and to choose longer campaign durations. Finally, based on readability indices, fraudsters are more likely to provide easier-to-read campaign pitches.

In sum, we identify which factors signal first-party enforcement and project quality, and our results illustrate their relevance in predicting subsequent fraudulent behavior.

In the second part of our analyses (*Platform-wide Consequences of Fraud*), we document that a large public crowdfunding scam can have an economically significant negative impact on concurrent projects. Therefore, a few incidences over a short period of time may cause a tremendously negative spillover effect.

We collect data on more than 270,000 campaigns from 2010 through 2018. As a result of Kickstarter “late” suspensions (which may signal weak related-party enforcement and inefficient platform pre-screening), the probability of reaching goal amount for campaigns launched around the same date is about 6.38% lower. On average, all else being equal, the pledged amount decreases by 9.6%.

Backers’ trust in platform integrity is especially vital because platform revenue is a percentage of raised amounts, leading to a potential agency problem. Backers may react negatively if they perceive suspended campaigns as not only first-hand evidence of weak legal enforcement, but also inefficient platform scrutiny. We highlight the importance of related-party enforcement and platform scrutiny before projects are posted, especially since platforms do not generally enforce accountability once funds are transferred to creators (e.g., by charging insurance fees proportional to campaign overcontributions).

Our paper is related to the growing literature on crowdfunding that, to date, has focused primarily on determinants of funding success (see, e.g., Agrawal et al., 2015; Ahlers et al., 2015; Belleflamme et al., 2013; Coakley & Lazos, 2021; Colombo et al., 2015; Mollick, 2014; Rossi et al, 2021; Vismara, 2016). Prior research has explored late deliveries (Mollick, 2014), project or firm failures (Hornuf et al., 2018; Signori & Vismara, 2018), factors affecting backer trust (Liang et al., 2019), mechanisms to deter misconduct (Belavina et al., 2020), and the impact of pro-social framing, altruism, and self-interest on crowdfunding success (André et al., 2017; Berns et al., 2020; Defazio et al., 2020). Other papers have examined the role of securities regulation in equity crowdfunding markets (Bradford, 2012; Hornuf & Schwienbacher, 2017), return on investment in equity crowdfunding (Hornuf et al., 2018; Signori & Vismara, 2018), and the dynamics of crowdfunding project support over time (Hornuf & Schwienbacher, 2018).

We contribute to the entrepreneurial finance literature by identifying specific campaign- and creator-related factors that correlate with fraudulent behavior in the crowdfunding market. We also document the negative effect of perceived weak platform scrutiny on the success of concurrent campaigns. Our study opens avenues for future research on crowdfunding fraud and its effects by developing and integrating new fraud detection models in an entrepreneurial finance setting (see, e.g., Allen et al., 2021; Perez et al., 2020).

The remainder of this paper is organized as follows. The next section develops our hypotheses. We then introduce the data and outline our methodology. The “Empirical Results” section presents univariate and multivariate empirical analyses, as well as several robustness checks. The final section concludes and discusses implications for research, practice, and policy.

## Theory and Hypotheses

Dupont and Karpoff (2019) explain the importance and fragility of trust in the process of economic exchange. They introduce a framework with three mechanisms to provide discipline, deter opportunistic behavior, and build sufficient trust.

The equity markets have shown that fraudulent activities can result in sharp declines in firm performance and share prices (Karpoff et al., 2008; Rezaee, 2005), but also in the collapse of entire market segments. In 1997, the market segment *Neuer Markt* was established on the German stock exchange, with the goal of financing innovative small and medium-sized growth companies. After a strong start, the segment reached a market capitalization of $234 billion (Hainz, 2018). However, several incidents of corporate fraud and misconduct eroded its reputation, and it was closed only 6 years after launch, down 90% from its market peak. Similarly, since crowdfunding is a new phenomenon, fraud cases can be very destructive and lead to spillover effects on future campaigns.

As mentioned earlier, the three legs of the “Trust Triangle” are: (1) first-party enforcement (personal ethics, integrity, culture); (2) related-party enforcement (market forces and reputational capital); and (3) third-party enforcement (laws, regulations, regulators). Legal enforcement by government agencies within the crowdfunding market has been relatively lax, and regulators have limited capacity for enforcement.^3^ Thus, project creators’ integrity and platform enforcement are of paramount importance in determining backers’ trust level. It is even more important since platform revenue is directly related to the amounts raised (usually a fixed percentage), and amounts over goal go to creators.

After a campaign ends, and funds are distributed, there is a risk that creators will cease working on the venture, or that they will use the funds to extract private benefits, creating a moral hazard problem (Hainz, 2018). This risk can be reduced by writing complete contracts, typically not feasible in this context, or by strengthening first- and related-party enforcement.

We focus on the first leg of the Trust Triangle and signals of project quality to develop Hypotheses 1–3. We aim to identify which creator and campaign characteristics are perceived as credible signals of first-party enforcement.

Economists and psychologists suggest various reasons why individuals engage in fraud. In a crowdfunding context, backers can analyze campaign pages on the platforms and draw their own expectations about quality and fraud probability. For example, they can read campaign descriptions and view campaign videos. All of this information clearly helps reduce asymmetric information, but it does not eliminate it. Fraudulent campaign creators, on the other hand, have a clear incentive to increase information asymmetries and hinder backers from distinguishing fraudulent projects. Therefore, it is necessary to identify creator and campaign features that can *ex ante* serve as signals of first-party enforcement and that are difficult or costly to mimic. We posit that fraudsters may implement symbolic actions to build trust and increase their chance of success (e.g., Zott & Huy, 2007).

In the realm of crowdfunding, we identify three broad themes where backers could theoretically identify signals of stronger first-party enforcement based on available information: (1) *creator(s)’ characteristics/background*, (2) *creator(s)’ social media affinity*, and (3) *campaign characteristics.*

Social psychologists argue that, even when people are acting dishonestly, they nevertheless remain concerned about maintaining a positive self-image (Gino et al., 2009; Jiang, 2013; Mazar et al., 2008). This brings us back to the first leg of the Trust Triangle, which suggests that personal ethics play an important role when campaign creators commit fraud. Mann et al. (2016) focus on non-violent crimes, and find that internal sanctions provide the strongest deterrents. The effect of legal sanctions was weaker and varied across countries. As a result, crowdfunding fraud may not only follow an economic calculation by a project creator, it may also reflect personal attitudes and reputation.

For example, we do not generally expect creators with a rich history of successful campaigns to suddenly launch fraudulent projects. As Diamond (1989) notes, creators build their reputations by engaging in the market more frequently, and could suffer large losses from misconduct. A history of multiple honest campaigns therefore signals experience, which may decrease the probability of future dishonest campaigns. Similarly, creators who have previously backed other crowdfunding projects are likely to believe in the overall idea of crowdfunding (Cumming et al., 2019b). This can make it difficult for them to reconcile the idea of leading a scam. However, we note that backing multiple projects is easier and less costly for fraudsters to mimic, as they can contribute small amounts to multiple campaigns to signal prior activity. In sum, we predict a negative relationship between crowdfunding fraud and the intensity with which a creator uses crowdfunding as a backer or a creator (see Hypothesis 1).

### Hypothesis 1

(Creator(s)’ Characteristics and Background) Crowdfunding fraudsters are less likely to have engaged in prior crowdfunding activities.

Backers can also easily screen creators’ social media activities. If personal ethics and a positive self-image are important, fraudsters may avoid the use of social media because it can facilitate fraud detection. Furthermore, an observable social media presence may indicate a creator has more to lose from cheating in terms of social connections, and could be subject to more intense monitoring. Similarly, to earlier work on the effect of media on corporate social responsibility (El Ghoul et al., 2019), we theorize that a social media presence can lower the risk of crowdfunding fraud. Moreover, early backers are often friends and family, which is a specific feature of non-equity crowdfunding (Agrawal et al., 2015; Colombo et al., 2015). Arguably, this could jeopardize the positive self-image of a campaign creator (Shalvi et al., 2015), and make committing outright fraud harder.

Lin et al. (2013) show that, in peer-to-peer lending, borrowers’ online friendships act as signals of credit quality and lead to a higher probability of successful funding. However, fraudsters may manipulate social media information, by, e.g., using phony Facebook pages. Hence, it is unclear whether elaborate fraudsters have fewer or more social media contacts, and how difficult it is to mimic this feature. The same is true for using fake links on campaign websites that lead to other fake websites purporting to support the trustworthiness of a campaign. This highlights the importance of the first leg of the Trust Triangle. Thus, we predict a negative correlation between social media use and fraud.

### Hypothesis 2

(Social Media Affinity) Crowdfunding fraudsters are less likely to have a social media presence, and to provide fewer external links.

Finally, *Campaign Funding and Reward Structure* and *Campaign Description Details*, which we group together as *Campaign Characteristics*, can provide credible signals of first-party enforcement and project quality (Spence, 1973). Shailer (1999) develops a theoretical model showing that the signals entrepreneurs provide to lenders (through information or actions) may assist them in allocating *ex ante* default probabilities based on lenders’ prior knowledge of group characteristics. We aim to identify and determine the value of such signals in crowdfunding, and gauge how they correlate with fraudulent behavior.

We observe that more confident creators restrict funding period duration because they believe their projects will be funded rapidly. But fraudsters are less likely to send credible signals of quality. So they may tend to extend funding period duration to raise as much capital as possible. Longer funding periods may make detection more likely, and increase the risk of not receiving funds. Consequently, it remains an empirical question as to whether a longer funding period reduces or increases the probability of fraud. But we believe short duration is a credible signal of project quality. We, therefore, derive Hypothesis 3.A as follows:

### Hypothesis 3.A

Crowdfunding fraudsters are more likely to implement longer funding periods.

While backers may detect fraud once, e.g., a creator fails to deliver a product, the ultimate prosecution of the scam may be the most important factor to a fraudster. As noted above, the smaller the amount invested by backers, the less likely they will be to engage in litigation. Consequently, fraudsters may simply target as many backers as possible who can only contribute small amounts. One common method is to create many different pledge categories, to smooth the way for small-size contributions. We, therefore, derive Hypothesis 3.B as follows:

### Hypothesis 3.B

Crowdfunding fraudsters are more likely to offer smaller minimum pledge allowance choices.

Research shows that perpetrating securities fraud in publicly traded firms is easier when confusion exists among investors (Fischel, 1982; Perino, 1998; Simmonds et al., 1992). Research on the manipulation of stock markets has long explored so-called “pump and dump” schemes. These schemes involve acquiring long positions in stocks, and then heavily promoting them online or by spoof trading (deleting orders before execution to keep up appearances of an active book). In this way, fraudsters encourage other investors to purchase the stocks at successively higher prices, and then they sell their own shares. In a similar way, crowdfunding fraudsters can heavily promote a campaign by offering many project enticements with various reward levels (Belleflamme et al., 2014; Mollick, 2014). Moreover, because they do not intend to ship anything or continue communicating with backers, they are not constrained by excess demand or other costs later on. We, therefore, derive Hypothesis 3.C as follows:

### Hypothesis 3.C

Crowdfunding fraudsters are more likely to offer a larger number of reward/pledge categories.

Finally, in crowdfunding markets, fraudulent campaign creators may try to increase information asymmetries to make it more difficult for backers to differentiate between scams and worthwhile projects. The main way to convey information about a project is through the description, which is normally a few thousand words (Cumming et al., 2019a). Crowdfunding fraudsters are, therefore, less likely to provide a professionally worded description in order to foster confusion and avoid detection. In contrast, professional entrepreneurs are likely to use campaign descriptions to signal quality.

It is complicated to accurately and professionally describe a product that does not exist. This is in line with findings by Siering et al. (2016), who show that linguistic and content-based cues in static and dynamic contexts can help predict fraudulent crowdfunding behavior. Parhankangas and Renko (2017) show that certain linguistic styles increase the probability of success of social campaigns, such as, e.g., those that make the campaign and creator(s) more relatable. Alternatively, simpler descriptions (without the need for specialized knowledge to understand them) may help fraudsters target a less educated crowd. We, therefore, derive Hypothesis 3.D as follows:

### Hypothesis 3.D

Crowdfunding fraudsters are more likely to use simply worded campaign descriptions (i.e., lower formal education required to understand the description on a first read).

Next, to develop Hypothesis 4, we focus on the second leg of the Trust Triangle. In general, reward-based crowdfunding platforms do not conduct sophisticated background checks or due diligence (in contrast to, e.g., equity crowdfunding platforms). However, Kickstarter employs a “Trust & Safety” team to assess campaigns, and they can recommend suspensions for rules violations. Note that suspended campaigns do not necessarily denote fraud. But the platform-wide consequences of observed incidences of misconduct detection, proxied for by campaign suspensions, are a priori not clear and thus worth investigating empirically.

For example, backers who observe campaigns being suspended may infer that related-party enforcement works. On the other hand, backers who learn that fraudulent campaign creators have already conducted many scams prior to suspension may infer the platform cannot ensure accountability and that the pre-screening process is inefficient. Hence, large-scale campaign suspensions that have already attracted many backers, raised large amounts of funds, and are close to their scheduled deadlines can substantially weaken backers’ confidence in their own fraud detection skills, as well as in related-party enforcement. Weaker trust may cause concurrent crowdfunding campaigns to face difficulties raising capital and achieving funding goals. We, therefore, derive Hypothesis 4 as follows:

### Hypothesis 4

(Platform-Wide Consequences of Fraud): Campaigns posted around a late and visible suspension of a successful crowdfunding project have a lower probability of success, tend to raise less funds, and attract fewer backers.

## Data

We divide our data collection into two parts. First, we categorize fraudulent campaigns, derive the respective fraud and matched non-fraud samples, and examine the factors associated with a higher likelihood of observing fraudulent behavior. Second, we construct our sample for studying platform-wide consequences of breaches of trust. Variable definitions are in Table 1.

**Table 1:** Variable definitions

Variable name	Description and calculation
Panel A (“Determinants of fraud” analyses)	
*Dependent variable*	
Fraud	Dummy variable indicating whether a campaign is associated with fraudulent activities that equals 1 if a fraudulent activity is detected for a campaign, and 0 otherwise
*Creator(s)’ characteristics/background*	
Creator-backed projects	Total number of projects backed by the creator since joining the platform
Creator-created projects	Total number of projects created by the creator since joining the platform
Waiting time (months)	Number of months between the day the creator joined the platform (Kickstarter) and the start date of the campaign
Formal name	Dummy variable that equals 1 if the project creator uses a formal profile name (i.e., [first name] [last name]), and 0 otherwise
Natural person	Dummy variable that equals 1 if the project creator is one/more than one natural person(s) as shown by the profile, and 0 otherwise
*Social media affinity*	
# External links	Total number of external links provided on campaign page
Facebook	Dummy variable that equals 1 if a personal Facebook/Facebook page is linked to the project’s web page on Kickstarter, and 0 otherwise
Facebook_Page	Dummy variable that equals 1 if a link to a Facebook page associated with the campaign is provided, and 0 otherwise
Facebook_Personal	Dummy variable that equals 1 if a link to a personal Facebook page associated with the campaign creator(s) is provided, and 0 otherwise
LinkedIn	Dummy variable that equals 1 if a link to a LinkedIn page of the creator(s) is provided, and 0 otherwise
Log (FB connections)	Natural logarithm of “the total friends of personal Facebook page linked to the project’s web page on Kickstarter, plus the total likes of Facebook page associated with the campaign.”
*Campaign funding and reward structure*	
Duration	Number of days between the campaign’s end date and start date
Min. pledge amount	Minimum amount (in USD) that a backer must pledge to participate and receive a certain reward/benefit (associated with the minimum pledge category)
No. of pledge categories	Total number of pledge categories. Each individual backer can pledge an amount associated with one of the categories and receive a specific reward/benefit
*Campaign description details*	
ARI	Automated Readability Index of the project description text. ARI equals $4.71\left(\frac{\text{Number of Characters}}{\text{Number of words}}\right)+0.5\times {\text{ASL}}-21.43$, where ${\text{ASL}}$ is average sentence length (i.e., number of words divided by number of sentences). ARI corresponds to a U.S. grade level; the lower the number, the easier the text is to understand
CL	Coleman–Liau index of the project description text. CL equals $5.88\left(\frac{\text{Number of Characters}}{\text{Number of words}}\right)-29.6\times {\text{ASL}}$, where ${\text{ASL}}$ is average sentence length (i.e., number of words divided by number of sentences). CL corresponds to a U.S. grade level; the lower the number, the easier the text is to understand
FKG	Flesch–Kincaid grade level of the project description text. FKG equals $0.39\times {\text{ASL}}+11.8\times {\text{ASW}}-15.59$, where ${\text{ASL}}$ is average sentence length (i.e., number of words divided by number of sentences), and ${\text{ASW}}$ is average number of syllables per word. FKG corresponds to a U.S. grade level; the lower the number, the easier the text is to understand
GF	Gunning Fog index of the project description text. The index equals $0.4 [{\text{ASL}}+100\left(\frac{\text{Number of complex words}}{\text{Total Number of words}}\right)]$, where ${\text{ASL}}$ is average sentence length (i.e., number of words divided by number of sentences), and ${\text{complex words}}$ are words with three or more syllables. The index estimates the years of formal education needed to understand the text on a first reading. The lower the number, the easier the text is to understand
Video pitch	Dummy variable that equals 1 if a video pitch is provided on the campaign’s page to describe the project, and 0 otherwise

Panel B (“Consequences of fraud” analyses)	
*Dependent variables (Success)*	
Funded	Dummy variable that equals 1 if the project reached its goal amount, and 0 otherwise
Log pledged	Natural logarithm of the project’s pledged amount in USD (regardless of the project’s success) + 1)
Log backers	Natural logarithm of the project’s total number of backers (regardless of the project’s success) + 1)
*Independent variables*	
Fraud period	Dummy variable that equals 1 if the campaign’s launch date is within ∓ 14 days of the suspension date of any of the identified suspended fraudulent campaigns and did not end before the announcement date of the suspended campaign, and 0 otherwise
Post-fraud	Dummy variable that equals 1 if the campaign’s launch date is within + 14 days of the suspension date of any of the identified suspended fraudulent campaigns (i.e., Post-Fraud), 0 if the campaign’s end date is within − 14 days of the suspension date (i.e., Pre-Fraud), and omitted otherwise
*Control variables*	
Duration	Number of days between the campaign’s end date and start date
Waiting time	Number of days between the campaign’s start date and the date the creator joined Kickstarter (i.e., created an account)
Featured	Dummy variable that equals 1 if the project is featured as “Projects We Love” by Kickstarter, and 0 otherwise
Log goal	Natural logarithm of the project’s goal amount in USD
Daily activity	Average daily number of projects that were “live” during campaign’s lifetime, divided by 1000

### Categorizing Fraudulent Behavior in Crowdfunding

A legal definition of fraud in crowdfunding is not simple to operationalize for an empirical study. This is because, to date, few cases have been tried by an ordinary judge. In a theoretical context, Belavina et al. (2020) note that platforms can leave backers exposed to two risks: (1) funds misappropriation, where entrepreneurs run away with backers’ money, and (2) performance opacity, where product specifications are misrepresented. Therefore, we focus on industrywide definitions of *detected fraud* and *suspected fraud* (see, e.g., *Crowdfund Insider*^4^ for an overview). We next describe our categorization of fraud in more detail based on media reported cases, resulting in a sample of 193 fraudulent campaigns.

The first category, *detected fraud*, includes (1) *pre-empted fraud*, when a supposedly fraudulent campaign is reported in the media but is either suspended by the platform or canceled by the creator before money is transferred to the creator’s account. Both typically result from backer complaints to the platform provider, or from online postings warning that the campaign carries a risk of fraud; and (2) *attempted fraud*, when fraud was not originally detected during the campaign’s funding period, and campaign creators obtain the amounts raised. In this case, after funding completion, backers may find that, e.g., creators misrepresented material facts, used intellectual property to which they do not hold legal rights, or that the project is an outright fake.

The second category, *suspected fraud*, occurs when a supposedly fraudulent campaign is reported in the media, and (1) three specific conditions (described below) are met simultaneously, or (2) the rewards are changed to the disadvantage of backers (condition 2). The three conditions are: Rewards are delayed by more than 1 year from the promised delivery date (condition 1a); the creators cease credible communications with backers, such as, e.g., updates on the campaign web page, for at least 6 months after a promised delivery date (condition 1b); and rewards are not delivered, and backers have been neither partially nor fully refunded as of December 31, 2018 (condition 1c).

Detecting campaigns where rewards were changed significantly can be accomplished by studying news articles on a particular campaign, or by reading comments posted by backers after rewards delivery. However, if delivery is overdue, it is more difficult to distinguish between fraudulent projects and those that failed or experienced normal unforeseen setbacks.

To overcome this problem, we categorize campaigns where rewards are delayed for at least 1 year after the delivery date as suspected fraud. But this is true *only* if (1) the creator has also not posted meaningful updates for at least 6 months after the originally promised date,^5^ (2) the promised reward is not delivered until the end of our observation period, and (3) backers were not at least partially refunded.^6^ To classify projects as suspected fraud, we tracked all campaigns until December 31, 2018. If any of the three criteria were met, we exclude the project from our suspected fraud sample.^7^ We acknowledge that extreme incompetence of project creators can be an alternative explanation for campaigns considered fraudulent. However, failing to provide explanations and updates is a form of serious misconduct.

Note that there are other forms of crowdfunding fraud that are outside the scope of this article, because they are not possible to detect in a comprehensive manner. These include so-called *stillborn fraud*, where a potential fraud campaign is rejected by the crowdfunding platform before it is launched. Fraud is also not necessarily limited to project creators; there have been cases of reported fraud by crowdfunding backers, and even by some platforms themselves.^8^

There is no commercial database available for fraud cases in crowdfunding, but our base media reports sample covers all actual and potential fraud campaigns reported on a website called Kickscammed (http://kickscammed.com). Kickscammed is an independent site where the crowd can report suspicious or fraudulent crowdfunding activities. It is not linked to Kickstarter.

Table 2 shows the steps in constructing our fraudulent campaign sample. As of April 30, 2016, we were able to identify and confirm 181 fraud cases for the 2010–2015^9^ period that were reported on Kickscammed and met our criteria for *detected* or *suspected fraud*. However, Kickscammed does not cover all instances of fraud on Kickstarter, so we complement our dataset with a news search using Google, Factiva, and LexisNexis. Our initial fraud dataset is, therefore, comprised of 200 fraudulent campaigns. After excluding 7 campaigns for which no data were available, our final sample consists of 193 fraudulent cases^10^ (see Table 2, Panel A).

**Table 2:** Derivation of fraudulent campaigns’ sample (“determinants of fraud” analyses)

Panel A	
Identified via	#
Kickscammed	181
News search	19
Total (initial cases)	200
Data not available	7
Total	193

Panel B		
Fraud category	Status	#
Detected fraud	Pre-empted	19
	Attempted	25
Suspected fraud	Rewards changed	5
	Rewards not delivered	144
Total		193

Panel C														
Category	2010	Vol.	2011	Vol.	2012	Vol.	2013	Vol.	2014	Vol.	2015	Vol.	No.	Total
Art			1	32,017			1	14,651					2	46,668
Comics			2	21,875			3	66,068					5	87,943
Crafts							1	13,359	2	31,115			3	44,474
Design	1	87,407	4	631,294	17	1,913,405	23	3,953,543	7	723,299	2	25,710	54	7,334,658
Fashion					1	94,279	2	25,648	2	114,318	1	10,371	6	244,616
Film and video			2	95,348	4	331,594	3	139,837	2	277,056			11	843,835
Food							4	208,084	1	13,355	1	20,780	6	242,219
Games			3	212,928	15	755,384	14	327,620	12	599,399	1	13,796	45	1,909,127
Music									2	18,452			2	18,452
Photography							1	8047					1	8047
Publishing	1	28,701							1	380,747			2	409,448
Technology					6	682,179	17	5,102,461	18	3,197,970	15	2,361,927	56	11,344,537
Total	2	116,108	12	993,462	43	3,776,841	69	9,859,319	47	5,355,710	20	2,432,583	193	22,534,023

Total amount raised	22,534,023
Failed	69,294
Detected	2,810,455
“Successful” fraudulent campaigns (Total amount: USD)	19,654,274

Panel D							
Country	2010	2011	2012	2013	2014	2015	Total
Australia						1	1
Canada		1	1	3	1	1	7
China					1		1
Germany						1	1
Hong Kong						1	1
Israel				1	1		2
Spain					1		1
United Kingdom				4	2	2	8
United States	2	11	42	61	41	14	171
Total	2	12	43	69	47	20	193

Panel B of Table 2 illustrates the differences in the number of identified fraud cases across categories. We mark 44 campaigns as *detected fraud* (19 “Pre-empted” and 25 “Attempted”), and 149 as *suspected fraud* (5 “Rewards Changed” and 144 “Rewards Not Delivered”).^11^ Our identified fraudulent campaigns (within the 2010–2015 period) seem low in comparison to the total number of projects on Kickstarter. This raises the question of whether we are only observing the tip of the iceberg, or whether fraud in crowdfunding is overly difficult to detect.

Following Hainz (2018), we find multiple major reasons why crowdfunding fraud may not be observable. Hainz (2018) underscores that (1) the efficiency of the crowd in detecting fraudulent campaigns is relatively high (most backers have experience from prior campaigns); (2) the effectiveness of platforms such as Kickstarter at filtering out fraudulent projects before they are posted is also relatively high; (3) non-reporting of fraudulent campaigns is highly likely, especially when a campaign is unsuccessful and no money has changed hands, because neither backers nor platform providers have a high incentive to report it; and (4) backers of successful but fraudulent campaigns may not bother to report fraud if they contributed only a small amount.

### Determinants of Fraud

In order to obtain a non-fraud control group with similar characteristics, we apply a propensity score matching (PSM) algorithm. We match our fraudulent campaigns only on campaign-related *demographic* characteristics (year, country, campaign category) and goal amount to ensure we do not select for other factors that could potentially explain fraudulent behavior.^12^

We implement a nearest-neighbor one-to-one fraud and non-fraud matching without a replacement option to ensure the random component of the sample. We then construct our sample for the main analyses. As a robustness check, we provide results based on one-to-one matches (with replacement option) and one-to-two matches (with and without replacement options). We consider 386 crowdfunding campaigns (193 one-to-one pairs of matched fraud and non-fraud campaigns) in our main analysis. We checked the campaign web pages of all non-fraud matches to ensure that none were suspected of fraud. We hand-collected information from Kickstarter on nineteen explanatory campaign variables, based on information from campaign web pages or social media pages associated with the campaign/creator.

### Platform-Wide Consequences of Fraud

Next, we study platform-wide consequences of breaches of trust. To this end, we use an event study-like setting to demonstrate whether late suspensions by Kickstarter, which we classify as large public scams based on four criteria, negatively affect the success of other campaigns launched around the same time. One challenge is to identify the “announcement date” that the fraud became visible to the community (i.e., potential backers). We use Kickstarter’s suspension dates for large successful campaigns associated with misconduct. Note again that there is no legal proof that suspended cases constitute outright fraud. If Kickstarter’s “Trust & Safety” team uncovers evidence that a campaign is in violation of its rules, the campaign is suspended, according to Kickstarter’s procedures.^13^

We scraped data on all suspended campaigns using the “Explore” function of Kickstarter, which resulted in 1760 campaigns with suspension dates between January 1, 2010, and September 30, 2018.^14^ Table 3 provides an overview within each main category for the respective year (Panel A) and pledged dollar volumes (Panel B).^15^ We use this population to determine the most severe and visible scam campaigns that attracted backers, as well as their “announcement dates,” as we describe below.

**Table 3:** Derivation of suspended campaigns’ sample (“consequences of fraud” analyses)

Panel A											
Num.	Main category	2010	2011	2012	2013	2014	2015	2016	2017	2018	Total (#)
1	Art	4	7		2	7	32	8	23	13	96
2	Comics					8	6	2	1	6	23
3	Crafts			1		10	35	6	13	5	70
4	Dance					6	6		1	2	15
5	Design		1	13	7	22	51	40	47	33	214
6	Fashion		4	4	5	21	42	15	23	19	133
7	Film & video	3	7	7	7	11	39	17	22	8	121
8	Food		7	1	3	17	60	23	25	16	152
9	Games	1	1	5	5	24	85	30	39	30	220
10	Journalism	1		1		9	23	7	5	5	51
11	Music	2	10	3	1	34	52	23	22	9	156
12	Photography	2	3	1	1	2	26	7	5	2	49
13	Publishing	1	3	4	1	5	22	7	19	3	65
14	Technology	3	2	9	6	48	99	72	92	46	377
15	Theater	1	2			4	9	1		1	18
	Total	18	47	49	38	228	587	258	337	198	1760

Panel B											
Num.	Main category	2010	2011	2012	2013	2014	2015	2016	2017	2018	Total (Vol. in USD 1000)
1	Art	> 0	0.34		10.50	0.55	14.17	2.21	25.25	2.46	55.46
2	Comics					5.19	1.74	0.92	> 0	4.93	12.77
3	Crafts			3.52		0.73	2.23	4.70	7.34	1.47	19.99
4	Dance					0.33	3.84		> 0	0.61	4.78
5	Design		> 0	149.26	73.77	162.62	664.84	956.65	1456.08	389.22	3852.44
6	Fashion		0.06	33.39	44.65	135.64	107.11	62.51	70.75	12.90	467.00
7	Film & video	0.05	0.60	48.38	41.63	65.25	28.38	30.51	0.88	16.26	231.92
8	Food		0.19	0.98	122.28	10.46	5.38	269.48	102.50	40.65	551.91
9	Games	> 0	0.07	20.34	107.80	114.68	57.99	11.54	76.89	173.78	563.09
10	Journalism	0.05		> 0		0.18	1.77	0.30	0.17	0.25	2.72
11	Music	> 0	0.10	21.37	5.74	1.60	5.49	3.30	6.59	8.87	53.05
12	Photography	> 0	> 0	> 0	> 0	> 0	1.60	6.23	2.73	1.94	12.49
13	Publishing	> 0	0.02	5.14	0.92	0.51	12.69	1.71	55.94	20.14	97.05
14	Technology	0.10	> 0	235.67	83.31	1708.16	5211.05	1266.12	1283.04	759.35	10,546.80
15	Theater	0.03	> 0			0.46	0.02	0.01		> 0	0.52
	Grand total	0.22	1.36	518.04	490.58	2206.34	6118.29	2616.17	3088.16	1432.85	16,472

	Panel C		
	Inclusion criteria	#	Sub-total
	Suspended campaigns sample	1760	–
1	More than 20% of campaign duration passed	− 859	901
2	Less than 1 week remaining to scheduled deadline	− 689	212
3	Number of backers = more than 1000	− 198	14
4	Pledged amount higher than USD 10,000	− 0	14
	Final number of suspended campaigns		14

Panel D						
Num.	Suspension date	Name	Main category	Goal (USD)	Pledged (USD)	# Backers
1	2013-06-13	KOBE RED—100% JAPANESE BEER FED KOBE BEEF JERKY	Food	2374	120,309	3252
2	2014-07-22	Areal	Games	50,000	64,928	1090
3	2015-08-05	TrackerPad—Sticky GPS Tracker Pads	Technology	155,194	80,651	1209
4	2015-08-11	Firestarter Survival Bracelet/Carabiner Paracord Keychain	Technology	10,000	477,462	9139
5	2015-10-12	The Skarp Laser Razor: twenty first Century Shaving	Technology	160,000	4,005,112	20,632
6	2016-01-27	TESLA—Self-rechargeable, Electronic Lighter	Technology	5000	118,693	3605
7	2016-10-19	λ Chair—The Advanced Art of Seating	Design	25,000	614,382	1531
8	2016-10-25	iLDOCK—Charge and Listen to iPhone 7 at the Same Time	Technology	5000	212,459	9895
9	2017-12-19	GARY 2.0: Earphones & Cables Automatic Organizer	Technology	6537	33,026	1650
10	2018-02-02	YT TOUCH | Fast Aerospace Aluminum Defrosting Tray	Design	10,000	212,632	4496
11	2018-02-24	Most Functional Duffel Bag Ever	Design	5764	108,781	1316
12	2018-05-09	Zōk | Restore Calmness and Serenity to the Mind and Body	Technology	10,500	56,673	1812
13	2018-06-22	amplify | The Ultimate Wireless Headphone Amplifier with DAC	Design	33,000	98,460	1220
14	2018-07-18	Overturn Rising Sands	Games	34,133	114,380	1093

We aim first to identify “late” suspensions. We posit that, if Kickstarter suspends a campaign in its early stages, this should be a positive signal to the crowd of related-party enforcement. Thus, we should not see a negative effect on other projects’ funding or on the market as a whole. Second, we aim to ensure that such announcements are as visible as possible to the crowd. We follow a two-step procedure to identify suspended campaigns (ensuring late suspension and visibility) with the highest negative platform-wide consequences, which can be regarded as large, public scams.

Late suspension criteria: First, at least 20% of the allegedly fraudulent campaign’s duration must have passed. Second, there must be < 1 week remaining to campaign end. These criteria ensure that the suspension was perceived as “late” in the crowdfunding community, and could in fact impact the funding success of other non-fraudulent campaigns. The first criterion reduces the total number of 1760 suspended campaigns by 859, and the second by 689, leaving us with 212 (see Table 3, Panel C).

Visibility criteria: Unfortunately, there is no direct measure of campaign visibility available, but we argue that it correlates highly with the number of pre-suspension backers in a campaign. The third criterion (that a suspended campaign must have attracted at least 1000 backers) is important, because 580 campaigns were suspended before a single backer contributed. If campaigns are suspended by Kickstarter before anyone can contribute, backers may believe related-party enforcement has worked. In that case, we do not expect to observe any negative impact on platform-wide funding activities.

We use another proxy for campaign visibility, pledged amount before campaign suspension. Therefore, we require, as a fourth criterion, that at least USD $10,000 have been contributed to a campaign before suspension. The criterion for the number of backers reduces the number of suspended campaigns by another 198, while the contribution requirement did not result in any further exclusions (see again Table 3, Panel C). In sum, based on the four criteria, we identified fourteen suspended campaigns that may have had a sizable negative platform-wide effect (see Table 3, Panel D).^16^

We then collect comprehensive data from Kickstarter for all campaigns with a goal amount of at least USD $100 (excluding very small donation-like campaigns), launched on or after January 1, 2010 and ending on or before December 31, 2018, and either successful/funded (reached goal amount) or unsuccessful/failed (did not reach goal amount).^17^ Our scraping procedure identified 271,971 unique campaigns within 15 main categories on Kickstarter.

Table 4 provides an overview, showing the number of launched campaigns for each year within the main categories (Panel A), their respective success rates (Panel B), and summary statistics (all values are converted to USD using Kickstarter’s static USD rate). It also shows the correlation matrix for all variables considered in the analyses of platform-wide consequences of fraud (Panel C).

**Table 4:** Overview of Kickstarter sample (2010–2018)

Panel A											
Num.	Main category	2010	2011	2012	2013	2014	2015	2016	2017	2018	Total (#)
1	Art	486	1656	2645	2761	3842	4449	3109	3429	3291	25,668
2	Comics	68	217	511	677	1128	1650	1640	1794	1798	9483
3	Crafts	23	62	143	295	1407	2090	1517	1307	915	7759
4	Dance	123	385	487	525	658	569	406	341	212	3706
5	Design	63	173	494	972	1989	3271	3906	4684	3779	19,331
6	Fashion	2	10	265	717	2544	3930	3245	3459	3043	17,215
7	Film & video	1020	2768	3975	4954	6177	6380	4535	3659	2799	36,267
8	Food	35	53	141	340	3582	4425	2759	2400	1729	15,464
9	Games	101	332	1311	2111	3649	4979	4777	5339	5126	27,725
10	Journalism	95	101	146	129	710	1188	678	497	332	3876
11	Music	1159	3242	5503	5277	5391	5864	3880	3350	2432	36,098
12	Photography	37	81	112	237	1554	1635	1068	832	554	6110
13	Publishing	307	1071	3042	4061	5582	5869	4467	4270	3188	31,857
14	Technology	140	240	458	1218	4393	6849	5345	4712	3011	26,366
15	Theater	21	67	82	273	1102	1309	916	742	534	5046
	Total	3680	10,458	19,315	24,547	43,708	54,457	42,248	40,815	32,743	271,971

Panel B											
Num.	Main category	2010	2011	2012	2013	2014	2015	2016	2017	2018	Total (#)
1	Art	51%	55%	51%	50%	37%	36%	40%	48%	57%	45%
2	Comics	54%	54%	50%	57%	57%	58%	65%	70%	77%	64%
3	Crafts	70%	71%	80%	68%	24%	23%	25%	28%	32%	29%
4	Dance	81%	76%	75%	74%	62%	51%	64%	63%	62%	66%
5	Design	56%	53%	48%	45%	38%	39%	48%	50%	55%	47%
6	Fashion	50%	50%	87%	69%	33%	26%	27%	34%	42%	34%
7	Film & video	46%	45%	40%	49%	43%	36%	41%	42%	47%	42%
8	Food	60%	64%	62%	58%	22%	21%	24%	27%	30%	25%
9	Games	38%	31%	27%	34%	30%	34%	41%	52%	60%	43%
10	Journalism	45%	43%	34%	43%	21%	18%	20%	24%	30%	24%
11	Music	45%	56%	59%	60%	52%	41%	47%	49%	56%	52%
12	Photography	43%	48%	46%	45%	25%	28%	40%	39%	47%	34%
13	Publishing	61%	58%	49%	44%	33%	30%	37%	39%	49%	39%
14	Technology	41%	49%	56%	48%	23%	22%	23%	25%	27%	25%
15	Theater	90%	82%	77%	66%	59%	58%	64%	61%	60%	61%
	Total	49%	53%	50%	51%	36%	32%	38%	42%	50%	41%

Panel C															
Num.	Variable	# Obs.	Mean	Std. dev	Min	Max	1	2	3	4	5	6	7	8	9
1	Funded	271,971	0.41	0.49	0.00	1.00	1								
2	Log pledged	271,971	5.99	3.23	0.00	11.96	0.67	1							
3	Log backers	271,971	2.81	1.88	0.00	7.52	0.71	0.93	1						
4	Fraud period	271,971	0.15	0.36	0.00	1.00	− 0.03	− 0.03	− 0.02	1					
5	Duration	271,971	33.16	11.55	8.00	60.00	− 0.14	− 0.06	− 0.07	− 0.02	1				
6	Waiting time	271,971	42.03	90.94	0.00	598.00	0.03	0.14	0.13	0.01	0.03	1			
7	Featured	271,971	0.10	0.30	0.00	1.00	0.27	0.33	0.39	− 0.02	− 0.03	0.06	1		
8	Log goal	271,971	8.55	1.59	5.02	12.61	− 0.23	0.12	0.1	0	0.21	0.13	0.12	1	
9	Daily activity	271,971	3.86	1.40	0.69	6.71	− 0.14	− 0.16	− 0.14	0.15	− 0.06	0.03	− 0.01	0.06	1

All non-dummy variables are winsorized at the 1% level on both sides

## Methods

We first specify a baseline regression model for the determinants of fraud analyses using three sets of characteristics: *creator’s characteristics/background*, *social media affinity*, and *campaign characteristics* (*campaign funding and reward structure*, as well as *campaign description details*). We apply a logistic regression model to examine the determinants of our dependent variable, *Fraud*, which equals 1 if the campaign is in our fraud sample, and 0 otherwise.

The non-fraud campaigns are based on a PSM approach using available *demographic* variables. This ensures that our control sample is not affected differently by national regulations, culture, project category, size, or time period of crowdfunding (Aggarwal et al., 2016; Attig et al., 2016; El Ghoul et al., 2016). The structure of our baseline logistic regression model is as follows:

1 $${Fraud \left(0/1\right)}_{i}= \alpha +{\sum\limits_{j} }{\gamma }_{j}\times {Creator(s){^{\prime}}\; Characteristics/Background}_{j}+{\sum\limits_{k} }{\xi }_{k}\times {Social\; Media\; Affinity}_{k}+{\sum\limits_{l} }{\varphi }_{l}\times {Campaign\; Funding\; and\; Reward\; Structure}_{l}+{\sum\limits_{m} }{\phi }_{m}\times {Campaign\; Description\; Details}_{m}+{\varepsilon }_{i}.$$

For each campaign *i*, the main explanatory variables are the *j* variables in the *creator(s)’ characteristics/background* block (*Creator-Backed Projects* and *Creator-Created Projects*)*.* The *k* variables in the *social media affinity* block include # *External Links* and *Facebook*. The *l* variables in the *campaign funding and reward structure* block include *Duration*, *Min. Pledge Amount*, and *No. of Pledge Categories*. Finally, the *campaign description details* block includes *m* variables, the *ARI*, and *Video Pitch*. We do not include year, country, or campaign category fixed effects because our samples have been initially matched and are balanced on those variables. See Bertoni et al. (2011), Grilli and Murtinu (2014), and Lee et al. (2015) for time variation and access to finance. We do use robust standard errors, which are one-way-clustered by campaign categories in all regressions, because residuals can be correlated within certain categories (Thompson, 2011).

We run several robustness checks, where we (1) use different nearest-neighbor matching procedures (one-to-one and one-to-two, with and without replacement options) for our main analysis, and (2) operationalize our theoretical concepts with different variables and alternative proxies for *creator(s)’ characteristics/background* (*Waiting Time (months)*, *Formal Name*, and *Natural Person*), *social media affinity* (*Facebook_Page*, *Facebook_Personal*, *LinkedIn*, *Log *(*FB Connections*)), and project description readability indices (*CL*, *FKG*, and *GF*).

Note that our model does not aim to specify the forecasted probability of a campaign being fraudulent for a given set of explanatory variables. This would be extremely difficult to achieve. King and Zeng (2001b) explain that, in a case–control design, where the fraction of failure in the data differs from that in the population, the *estimated probabilities* (i.e., forecasts) are biased and need *prior correction*. King and Zeng (2001a) posit that, for logit models with unknown sampling probability, as in our set-up, the *constant term* is biased but the parameter estimates remain largely unbiased. Therefore, prior correction is applied only to the constant term. However, the calculation of the correction term, which is subtracted from the estimated constant term, requires knowledge of the underlying probability of fraud in the population. This is not known to us, because false negatives in the population may prevent us from calculating the correction. Thus, we are only interested in the coefficients of the independent variables that have been shown to be unaffected and that are generalizable to the population (King & Zeng, 2001a, 2001b).

Second, we present the methodology related to our platform-wide consequences of fraud analyses. We require a goal amount of at least USD $100 to avoid micro campaigns. To determine whether the dynamics differ for campaigns that are more likely to be related to entrepreneurial activities, we require a goal amount of at least USD $10,000, and we repeat the analyses (see Mollick and Nanda (2015) for a similar argument). The structure of our logistic (and OLS) regression model is as follows:

2 $${Success}_{i}= {\beta }_{0}+{\beta }_{1,a}\times {Fraud\; Period}_{i}+{\beta }_{1,b}\times {Post\; Fraud}_{i}+{\beta }_{2}\times {Duration}_{i}+{\beta }_{3}\times {Waiting\; Time}_{i}+{ \beta }_{4}\times {Featured}_{i}+{ \beta }_{5}\times {Log\; Goal}_{i}+{ \beta }_{6}\times {Daily\; Activity}_{i,a}+{\phi }_{a,b}+{\varphi }_{a,b}+{\lambda }_{a}+{\theta }_{a}+{\xi }_{a}+{\varepsilon }_{i},$$

for each campaign *i*, *Success* represents the dummy variable *Funded* (Logistic), the variable *Log Pledged* (OLS), or the variable *Log Backers* (OLS). Our main variable of interest is (1) the dummy variable *Fraud Period*, which equals 1 if the campaign’s start date is within 14 days ($\mp$ 14) of the late suspension announcement, and 0 otherwise, or (as an alternative proxy) (2) the dummy variable *Post-Fraud*, which equals 1 if the campaign’s start date is within 14 days of the late suspension announcement, and 0 if it ended before that (we omit campaigns with other start/end dates).

If Hypothesis 4 is supported, we expect to find negative coefficients for ${\beta }_{1,a}$ and ${\beta }_{1,b}$ for all three success measures. We control for the three main variables (i.e., *Duration*, *Featured*, and *Log Goal*), which are also used in Mollick (2014) and have a significant influence on campaign success, plus *Waiting Time* to proxy for a creator’s experience on the platform. We also introduce a new control variable, *Daily Activity*, to proxy for the level of competition while a project is live.

Classifying a campaign as posted within a fraud period is not as straightforward as for an ordinary event study. A campaign suspension is not typically a “1-day” event, because, e.g., campaigns launched before the suspension date that have a deadline scheduled for after it are affected by the suspension, as are those launched closely afterward. We define a dummy variable “Fraud Period” for each of the 271,971 campaigns that equals 1 if the campaign is launched within 14 days before/after any of the identified suspension dates.^18^ We choose 14 days because most campaigns have a duration of about 30 days. We change the definition from $\mp$ 7 to $\mp$ 29 days, instead of $\mp$ 14, for the robustness checks.

When using the classification *Fraud Period* to identify campaigns most likely to be affected by a suspension announcement, we include a series of fixed effects: campaign category ($\phi$), year (2010–2018) ($\varphi$), month of year (January–December) ($\lambda$), day of month (first day to last day of respective month) ($\theta$), and day of week (Monday–Sunday) ($\xi$) to capture dynamics in different categories, as well as any time effect that may influence crowdfunding (and platforms) in certain years, months, or days. We also include *Daily Activity* (average daily number of “live” projects during a campaign’s lifetime). This variable captures the effects on campaign success that are directly related to platform activity but have not been picked up by the series on fixed effects. This is highly important. Intuitively, we expect that competition intensity (the number of live campaigns on the platform) is inversely correlated with campaign success (see Chen 2021 for empirical evidence).

For the alternative classification, *Post-Fraud*, we determine a direct *pre*- vs. *post*-fraud comparison in success levels of a subsample of the projects posted around the identified dates. We also include fixed effects: campaign category ($\phi$) and year (2010–2018) ($\varphi$). We use clustered robust standard errors based on campaign categories in all regressions. The alternative classification *Post-Fraud* allows for a more direct comparison because it has fewer observations and substantially reduces the need to control for periodic fixed effects.

## Empirical Results

We use two different samples to study (1) determinants of fraud (credible signals of first-party enforcement), and (2) platform-wide consequences of perceived weak related-party enforcement. We then check for robustness by examining the impact of signals of first-party enforcement (and project quality) on project success, especially when related-party enforcement (platform scrutiny) is perceived to be weak.

For studying “determinants of fraud,” it is important to have a high level of certainty that the identified campaigns are fraudulent, or at least largely perceived as such. This is why we do not include all campaigns reported on Kickscammed or in the media in our dataset. Instead, we check outcomes, e.g., whether the promised product was finally delivered or any communication attempted, to distinguish “failed” from “fraudulent” projects. To study measurable platform-wide consequences, it is critical to identify campaigns suspended later than expected, of larger size, with higher numbers of backers, and with higher pledged amounts in order to ensure that other backers (besides those directly affected) could have reacted to a suspension announcement. Therefore, we conducted the filtering process described previously to gauge which campaigns had the most damaging effects on the market.

### Determinants of Fraud

We begin by discussing our results in a univariate setting, and then focus on multivariate analyses to include multiple possible determinants of fraud simultaneously. Table 5 (Table 10 in the Appendix) shows the descriptive statistics (correlation matrix) for the explanatory variables.

**Table 5:** Summary statistics (“determinants of fraud” analyses)

Variable	# Obs.	Mean	Std. dev.	Min	Max
*1. Creator(s)’ characteristics/background*					
(1) Creator-backed projects	386	12.63	21.55	0	109
(2) Creator-created projects	386	0.96	1.90	0	9
(3) Waiting time (months)	379	10.15	11.29	0	42
(4) Formal name	386	0.42	0.49	0	1
(5) Natural person	386	0.49	0.50	0	1
*2. Social media affinity*					
(6) # External links	386	1.81	1.34	0	5
(7) Facebook	386	0.58	0.49	0	1
(8) Facebook_Page	386	0.24	0.43	0	1
(9) Facebook_Personal	386	0.47	0.50	0	1
(10) LinkedIn	386	0.03	0.17	0	1
(11) Log (FB connections)	213	6.71	1.37	3.04	9.48
*3.1. Campaign funding and reward structure*					
(12) Duration	386	34.36	10.01	15	60
(13) Min. pledge amount	386	10.12	18.50	1	99
(14) No. of pledge categories	386	12.60	6.79	4	36
*3.2. Campaign description details*					
(15) ARI	386	11.39	2.19	7.30	16.90
(16) CL	386	12.32	1.88	8.94	16.77
(17) FKG	386	9.21	1.74	6	13.4
(18) GF	386	8.63	1.22	6.40	11.60
(19) Video pitch	386	0.93	0.26	0	1

All non-dummy variables are winsorized at the 2.5% level on both sides

Table 6 shows the results for a difference in means *t*-test about how the fraud sample differs from non-fraud matched campaigns on our main explanatory variables. Note that fraudsters tend to have fewer backed projects (about five), and create fewer projects (about one). They also have a shorter period between date of account opening on Kickstarter and launch date (three to four months). In line with Hypothesis 1, the univariate comparison provides initial evidence that fraudsters are less likely to have engaged in prior crowdfunding activity.

**Table 6:** Mean differences between fraud and matched sample (“determinants of fraud” analyses)

Variable	Fraud		Non-fraud		*t*-test for difference in means
	# Obs.	Mean	# Obs.	Mean	
*1. Creator(s)’ characteristics/background*					
(1) Creator-backed projects	193	10.12	193	15.14	− 5.02**
(2) Creator-created projects	193	0.60	193	1.32	− 0.73***
(3) Waiting time (months)	187	8.31	192	11.94	− 3.63***
(4) Formal name	193	0.41	193	0.43	− 0.02
(5) Natural person	193	0.49	193	0.49	0.00
*2. Social media affinity*					
(6) # External links	193	1.47	193	2.14	− 0.67***
(7) Facebook	193	0.50	193	0.66	− 0.16***
(8) Facebook_Page	193	0.18	193	0.31	− 0.12***
(9) Facebook_Personal	193	0.40	193	0.54	− 0.15***
(10) LinkedIn	193	0.04	193	0.03	0.01
(11) Log (FB connections)	91	6.56	122	6.82	− 0.26
*3.1. Campaign funding and reward structure*					
(12) Duration	193	35.52	193	33.21	2.31**
(13) Min. pledge amount	193	9.61	193	10.62	− 1.01
(14) No. of pledge categories	193	13.37	193	11.82	1.55**
*3.2. Campaign description details*					
(15) ARI	193	11.13	193	11.65	− 0.52**
(16) CL	193	12.17	193	12.47	− 0.30
(17) FKG	193	9.06	193	9.36	− 0.31*
(18) GF	193	8.52	193	8.73	− 0.21*
(19) Video pitch	193	0.93	193	0.93	− 0.01

All non-dummy variables are winsorized at the 2.5% level on both sides

***, **, and * indicate statistical significance at the 1%, 5%, and 10% levels, respectively

In accordance with Hypothesis 2, we find that the number of external links is negatively related to fraud. It seems that external links serve a kind of certification role. Thus, more links imply higher reputational capital that can be lost in the case of a fraudulent campaign. We also find that fraudsters are less present or active on Facebook (66% of non-fraud campaigns link to Facebook, compared to only 50% of fraudulent campaigns). The results remain consistent if we examine personal Facebook accounts and Facebook pages separately.

In terms of *campaign characteristics*, and in accordance with Hypothesis 3, we find that campaign durations tend to be longer for fraudulent campaigns, with an average difference of 2 days. This small variation may be because Kickstarter generally recommends 30 days or less,^19^ and most projects follow that advice. We note that fraudulent campaigns provide more pledge categories, and their descriptions are easier to read. They can also be interpreted as less sophisticated, because most readability measures correspond to the number of years of formal education needed to understand the text upon first reading. The rationale behind this finding is that fraudsters are either targeting a wider and presumably less educated crowd, or they put less effort into the descriptions. However, we find no differences between fraud and non-fraud campaigns’ use of video pitches. This may be because creators are aware that video pitching is encouraged by platforms and can strongly impact the probability of successful fundraising, as per previous research (e.g., Mollick, 2014).

We turn next to our baseline model, which uses multivariate regressions to evaluate correlations among the three blocks of explanatory variables with fraud—*creator(s)’ characteristics/background*, *social media affinity*, and *campaign characteristics*. Table 7 summarizes our results for the determinants of fraud in Eq. (1). We consider all the main explanatory variables simultaneously. The means of the Variance Inflation Factors (VIFs) range from 1.10 to 1.12. Since they are well below the critical value of 5, there is no indication of multicollinearity (see Kutner et al., 2005).

**Table 7:** Multivariate analysis of determinants of fraud

	(1)	(2)	(3)	(4)
*1. Creator(s)’ characteristics/background*				
(1) # Creator-backed projects	− 0.008	− 0.002	− 0.009**	− 0.010
	(− 1.40)	(− 0.38)	(− 2.11)	(− 1.19)
(2) # Creator-created projects	− 0.183**	− 0.203*	− 0.124**	− 0.158**
	(− 2.23)	(− 1.86)	(− 1.98)	(− 2.30)
*2. Social media affinity*				
(3) # External links	− 0.355***	− 0.425***	− 0.428***	− 0.454***
	(− 5.64)	(− 7.53)	(− 7.50)	(− 10.59)
(4) Facebook	− 0.606**	− 0.672***	− 0.279	− 0.285
	(− 2.31)	(− 3.42)	(− 1.44)	(− 1.29)
*3.1. Campaign funding and reward structure*				
(5) Duration	0.031***	0.026**	0.026***	0.022**
	(3.37)	(2.52)	(2.61)	(2.12)
(6) Min. pledge amount	0.001	0.001	0.001	0.002
	(0.13)	(0.12)	(0.28)	(0.27)
(7) No. of pledge categories	0.054***	0.075***	0.042***	0.045***
	(3.09)	(8.28)	(2.59)	(3.66)
*3.2. Campaign description details*				
(8) ARI	− 0.116***	− 0.079*	− 0.131***	− 0.123***
	(− 3.09)	(− 1.92)	(− 3.76)	(− 3.15)
(9) Video pitch	0.156	0.019	− 0.117	− 0.055
	(0.60)	(0.07)	(− 0.29)	(− 0.10)
Constant	0.675*	0.817	0.610	1.182
	(1.66)	(1.15)	(0.94)	(1.51)
Replacement	No	Yes	No	Yes
# of Matching campaigns	1:1	1:1	1:2	1:2
Mean VIF	1.11	1.12	1.10	1.10
Maximum VIF	1.24	1.26	1.24	1.24
Observations	386	321	579	424
Pseudo *R*^2^	0.118	0.133	0.105	0.112

This table applies logistic regressions to analyze the determinants of fraud, where the dependent variable equals 1 if the campaign is fraudulent, and 0 otherwise. The coefficients are the logs of the odds ratios. All non-dummy variables are winsorized at the 2.5% level on both sides. Robust standard errors are one-way-clustered by campaign category. t-statistics are in parentheses

***, **, and * indicate statistical significance at the 1%, 5%, and 10% levels, respectively

Our main analysis is in Specification (1), for which the matched non-fraud campaigns are determined by using a one-to-one PSM nearest-neighbor matching method without replacement. For robustness checks, we also show results with replacement (Specification (2)), and for a one-to-two matching method with and without replacement (Specifications (3) and (4)).

*No. of Creator-Backed Projects* is negatively correlated with fraud. The coefficient remains stable throughout the specifications, but is only statistically significant in Specification (3). We also find that *No. of Creator-Created Projects* is negatively related to fraud; the coefficient is statistically significant throughout all specifications. This supports Hypothesis 1, that project creators with higher levels of prior crowdfunding experience are less likely to conduct fraudulent campaigns. It also confirms that backing multiple projects is easier to mimic as a signal for fraudsters than previously created projects.

As shown in Table 7, our main explanatory variables*—# External Links* and *Facebook—*in the s*ocial media affinity* block have a strongly negative relationship with fraud. Therefore, campaigns with either a Facebook page or a personal Facebook account are about 45% (= EXP (− 0.606) − 1) less likely to be fraudulent than their matches (significant at a 5% level—Specification (1)). The number of external links provided on the campaign website (e.g., a YouTube video associated with the campaign, a LinkedIn profile, a start-up’s web page) has a strongly negative correlation with the probability of a campaign being fraudulent. Overall, the results support Hypothesis 2, that fraudsters tend to be less present on social media and provide fewer external links.

Furthermore, in accordance with Hypothesis 3, we find that many *campaign characteristics* are related to the probability of observing fraudulent behavior. For example, fraudulent campaigns tend to *ex ante* choose longer funding durations (Hypothesis 3.A). This also comports with the signaling argument that high-quality campaigns choose shorter durations to signal quality and confidence in attaining funded. We find no statistical significance for *Min. Pledge Amount* (Hypothesis 3.B). This may be because most reward-based crowdfunding campaigns offer small amounts as minimum contributions for non-monetary payoffs, and thus campaigns do not substantially differ on this variable. Our results also show that the number of pledge categories has a significantly positive relationship with fraud. This provides further evidence for Hypothesis 3.C, that crowdfunding fraudsters are more likely to offer a higher number of reward levels.

Finally, Table 7 shows that project descriptions of fraudulent campaigns tend to have lower automated readability indexes (ARI). ARI is an approximate representation of the number of formal years of education needed to comprehend the text on a first reading. A one-level ARI increase from the average score of eleventh grade (U.S. grade level) to twelfth grade decreases the probability of the campaign being in the fraudulent subsample by about 10.5% (= EXP (− 0.116) − 1). This supports Hypothesis 3.D, that fraudsters may target a less educated crowd by using simpler language, or that they do not bother fine-tuning their campaign descriptions. We find no statistically significant effect of *Video Pitch* on fraud. This may be because more than 93% of our 386 cases used video pitches.

We check the robustness of our “determinants of fraud” results by using alternative proxies or complementary explanatory variables in Table 8. To avoid multicollinearity, or interdependent definitions across variables, we do not estimate models with all variables simultaneously. We examine each variable separately, but retain the main explanatory variables from the other blocks as “controls.” Control 1 (creator(s)’ characteristics/background) includes *Creator-backed Projects* and *Creator-created Projects*; Control 2 (social media affinity) includes *# External Links* and *Facebook*; Control 3 (campaign characteristics) includes *Duration, Min. Pledge Amount, No. of Pledge Categories, ARI*, and *Video Pitch*.

**Table 8:** Multivariate analysis of determinants of fraud (robustness check)

Panel A					
	(1)	(2)	(3)	(4)	(5)
(1) Creator-backed projects	− 0.013**				
	(− 2.48)				
(2) Creator-created projects		− 0.215***			
		(− 2.65)			
(3) Waiting time (months)			− 0.026**		
			(− 2.45)		
(4) Formal name				− 0.078	
				(− 0.40)	
(5) Natural person					− 0.012
					(− 0.07)
Constant	0.308	0.727*	0.408	0.370	0.309
	(0.87)	(1.85)	(1.12)	(0.87)	(0.83)
Control 1	No	No	No	No	No
Control 2	Yes	Yes	Yes	Yes	Yes
Control 3	Yes	Yes	Yes	Yes	Yes
Mean VIF	1.07	1.06	1.06	1.08	1.08
Maximum VIF	1.12	1.11	1.12	1.14	1.15
Observations	386	386	379	386	386
Pseudo *R*^2^	0.105	0.115	0.105	0.094	0.094

Panel B						
	(1)	(2)	(3)	(4)	(5)	(6)
(1) # External links	− 0.402***					
	(− 6.06)					
(2) Facebook		− 0.792***				
		(− 3.00)				
(3) Facebook_Page			− 0.864***			
			(− 6.87)			
(4) Facebook_Personal				− 0.650**		
				(− 2.46)		
(5) LinkedIn					− 0.017	
					(− 0.05)	
(6) Log (FB connections)						− 0.108
						(− 0.88)
Constant	0.689*	0.525	0.381	0.642	0.520	1.427
	(1.69)	(1.25)	(0.86)	(1.51)	(1.18)	(1.57)
Control 1	Yes	Yes	Yes	Yes	Yes	Yes
Control 2	No	No	No	No	No	No
Control 3	Yes	Yes	Yes	Yes	Yes	Yes
Mean VIF	1.10	1.10	1.09	1.09	1.09	1.11
Maximum VIF	1.24	1.23	1.23	1.23	1.23	1.28
Observations	386	386	386	386	386	213
Pseudo *R*^2^	0.105	0.087	0.085	0.080	0.064	0.121

Panel C								
	(1)	(2)	(3)	(4)	(5)	(6)	(7)	(8)
(1) Duration	0.027***							
	(2.98)							
(2) Min. pledge amount		− 0.004						
		(− 0.64)						
(3) No. of pledge categories			0.053***					
			(2.90)					
(4) ARI				− 0.103***				
				(− 3.27)				
(5) CL					− 0.088**			
					(− 2.12)			
(6) FKG						− 0.101**		
						(− 2.40)		
(7) GF							− 0.105**	
							(− 2.26)	
(8) Video pitch								0.149
								(0.48)
Constant	0.209	1.133***	0.546*	2.234***	2.156***	2.008***	1.980***	0.954***
	(0.67)	(5.27)	(1.91)	(6.40)	(4.48)	(5.95)	(3.99)	(3.22)
Control 1	Yes	Yes	Yes	Yes	Yes	Yes	Yes	Yes
Control 2	Yes	Yes	Yes	Yes	Yes	Yes	Yes	Yes
Control 3	No	No	No	No	No	No	No	No
Mean VIF	1.13	1.11	1.12	1.11	1.11	1.11	1.11	1.11
Maximum VIF	1.22	1.19	1.19	1.18	1.18	1.18	1.18	1.19
Observations	386	386	386	386	386	386	386	386
Pseudo *R*^2^	0.089	0.080	0.097	0.087	0.083	0.084	0.081	0.079

This table applies logistic regressions to analyze the determinants of fraud using alternative specifications and proxies, where the dependent variable equals 1 if the campaign is fraudulent, and 0 otherwise. The coefficients are the logs of the odds ratios*.* All non-dummy variables are winsorized at the 2.5% level on both sides. Robust standard errors are one-way-clustered by campaign category. t-statistics are in parentheses

***, **, and * indicate statistical significance at the 1%, 5%, and 10% levels, respectively

First, within the *creator(s)’ characteristics/background* block, we test for a relationship between a formal name profile and a natural person profile and the likelihood of a fraudulent campaign. We find no statistically significant relationship. This is attributable to the fact that, on Kickstarter, for example, project creators must verify their identities through an automated process. This information appears on their profiles (although not necessarily as their “profile names”).^20^ However, similarly to backing and creating crowdfunding campaigns, we find that our non-fraud sample creators have, on average, been members of the platform for longer periods of time.

We also test for the influence of social media connections. To avoid having outliers drive our results, we take the natural logarithm of number of connections, defined as the number of friends of a personal Facebook page associated with the campaign creator(s), plus the total number of likes. Despite finding a negative relationship between *Log (FB Connections)* and the probability of observing fraud, there is no statistically significant separate impact on fraudulent activity. We note that fraud campaigns may be using fake profiles to increase their numbers of “friends” or “likes” in order to mislead potential backers.

Furthermore, within the *campaign description details*, we identify a significantly negative relationship between ARI and fraud. That is, the probability that the campaign is in our fraudulent sample is higher when the project description is easier to understand. We further check for robustness by using three alternative measures of text readability. As Table 8, Panel C, shows, the Coleman–Liau index (*CL*), the Gunning Fog index (*GF*), and the Flesch–Kincaid Grade level index (*FKG*) all exhibit significantly negative correlations with fraudulent activity. This further validates our inferences.

In sum, our results remain robust to using alternative proxies for prior crowdfunding activity, social media affinity, and readability indices.

### Platform-Wide Consequences of Fraud

Table 9 presents the results of multivariate logistic and OLS regressions for our measures of success from Eq. (2). We test for platform-wide consequences of suspended large, public scam campaigns. In Panels A and B, Specifications (1)–(3) include Kickstarter campaigns with a goal amount of at least USD $100; Specifications (4)–(6) show results for campaigns with a goal amount of at least USD $10,000. We analyze the determinants of success measured by *Funded* (logistic regression; coefficients are the logs of the odds ratios), *Log Pledged* (OLS regressions), and *Log Backers* (OLS regressions). Campaigns affected by suspension announcements are classified with the dummy variable Fraud Period (Panel A) or Post-Fraud (Panel B).^21^

**Table 9:** Multivariate analysis of platform-wide consequences of fraud

Panel A						
	(1)	(2)	(3)	(4)	(5)	(6)
	Funded	Log pledged	Log backers	Funded	Log pledged	Log backers
	Goal amount > 99 USD			Goal amount > 9999 USD		
Fraud period	− 0.066***	− 0.096***	− 0.053***	0.014	− 0.056*	− 0.023
	(− 4.60)	(− 4.54)	(− 4.82)	(0.63)	(− 2.00)	(− 1.50)
Duration	− 0.020***	− 0.019***	− 0.012***	− 0.018***	− 0.018***	− 0.011**
	(− 6.64)	(− 5.02)	(− 4.10)	(− 3.35)	(− 3.46)	(− 2.70)
Waiting time	0.001***	0.003***	0.002***	0.001***	0.004***	0.002***
	(9.10)	(10.33)	(12.01)	(6.34)	(9.52)	(12.41)
Featured	2.397***	3.244***	2.280***	2.623***	4.012***	2.787***
	(19.14)	(16.09)	(19.65)	(18.31)	(17.63)	(24.58)
Log goal	− 0.385***	0.174***	0.076***	− 0.729***	− 0.423***	− 0.258***
	(− 23.11)	(6.38)	(4.21)	(− 10.74)	(− 5.33)	(− 7.12)
Daily activity	− 0.188***	− 0.334***	− 0.169***	− 0.161***	− 0.369***	− 0.187***
	(− 8.65)	(− 10.74)	(− 8.92)	(− 5.28)	(− 10.47)	(− 7.94)
Constant	3.441***	4.954***	2.159***	6.128***	10.127***	4.972***
	(13.61)	(16.75)	(13.95)	(8.52)	(10.82)	(10.89)
Category FE	Yes	Yes	Yes	Yes	Yes	Yes
Year FE	Yes	Yes	Yes	Yes	Yes	Yes
Month of year FE	Yes	Yes	Yes	Yes	Yes	Yes
Day of month FE	Yes	Yes	Yes	Yes	Yes	Yes
Day of week FE	Yes	Yes	Yes	Yes	Yes	Yes
Observations	271,971	271,971	271,971	98,702	98,702	98,702
Mean VIF	1.04	1.04	1.04	1.02	1.02	1.02
Maximum VIF	1.09	1.09	1.09	1.03	1.03	1.03
Adjusted *R*^2^		0.202	0.260		0.266	0.336
Pseudo *R*^2^	0.160			0.216		

Panel B						
	(1)	(2)	(3)	(4)	(5)	(6)
	Funded	Log pledged	Log backers	Funded	Log pledged	Log backers
	Goal amount > 99 USD			Goal amount > 9999 USD		
Post-fraud	− 0.117***	− 0.202***	− 0.095***	− 0.115***	− 0.244***	− 0.102**
	(− 5.38)	(− 6.72)	(− 5.65)	(− 2.61)	(− 3.59)	(− 2.56)
Duration	− 0.019***	− 0.020***	− 0.012***	− 0.019**	− 0.021**	− 0.012*
	(− 4.54)	(− 3.99)	(− 3.13)	(− 2.37)	(− 2.83)	(− 2.08)
Waiting time	0.001***	0.003***	0.002***	0.001***	0.004***	0.002***
	(8.34)	(11.50)	(14.80)	(3.61)	(11.40)	(12.97)
Featured	2.637***	3.462***	2.417***	2.842***	4.250***	2.933***
	(17.84)	(14.23)	(18.94)	(16.00)	(15.47)	(22.39)
Log goal	− 0.360***	0.156***	0.070***	− 0.740***	− 0.513***	− 0.314***
	(− 27.64)	(4.96)	(3.26)	(− 8.30)	(− 6.24)	(− 6.71)
Constant	3.456***	5.711***	2.669***	7.079***	12.548***	6.637***
	(21.74)	(20.38)	(15.06)	(8.43)	(16.97)	(17.90)
Category FE	Yes	Yes	Yes	Yes	Yes	Yes
Year FE	Yes	Yes	Yes	Yes	Yes	Yes
Observations	37,255	37,255	37,255	13,978	13,978	13,978
Mean VIF	1.01	1.01	1.01	1.01	1.01	1.01
Maximum VIF	1.03	1.03	1.03	1.03	1.03	1.03
Adjusted *R*^2^		0.208	0.271		0.270	0.340
Pseudo *R*^2^	0.167			0.220		

This table analyzes the determinants of *Success* measured by *Funded* (logistic regression; coefficients are the logs of the odds ratios), *Log Pledged* (OLS regressions), and *Log Backers* (OLS regressions). All non-dummy variables are winsorized at the 1% level on both sides. Robust standard errors are one-way-clustered by campaign category. *t*-statistics are in parentheses

***, **, and * indicate statistical significance at the 1%, 5%, and 10% levels, respectively

Panel A shows that the coefficient of *Fraud Period* is negative and highly statistically significant for the entire sample, including all campaigns with a goal amount of more than $100 (see Specifications (1)–(3)). In Panel B, we follow a stricter approach, and compare campaigns that ended within 14 days of the announcement (*Post-Fraud* = 0) with those begun within 14 days afterward (*Post-Fraud* = 1). This allows for a more direct comparison while requiring fewer observations. It also substantially reduces the need to control for *Daily Activity* and the sets of “periodic fixed effects” used in Panel A. This is because the pre- and post-fraud campaigns were launched around the same time, which mitigates any concerns about procyclicality.

Overall, the results in Table 9 provide strong empirical support for Hypothesis 4, that the occurrence of fraudulent campaigns and their visibility to potential backers have far-reaching consequences for the success (success probability, number of backers, and funds raised) of concurrent crowdfunding campaigns that begin around suspension dates. Panel A, Specification (1), shows a 6.38% lower probability of funding for campaigns posted within 14 days before/after one of our fourteen identified Kickstarter campaign suspensions (= EXP (− 0.066) − 1), all else being equal (see the coefficient on *Fraud Period*). In Specifications (2) and (3), the pledged amounts (number of project backers) also decreased in an economically meaningful way. The predicted pledged amount in Specification (2) (predicted number of backers in Specification (3)) is approximately 9.6% (5.3%) lower for projects posted within the fraud period (see again the coefficient on *Fraud Period*).

For example, considering the average pledged amount of approximately USD $11,000,^22^ campaigns posted within a fraud period lose about USD $1000 of their pledged amounts. The real effect is greater for raised amounts that are actually transferred, because within-fraud period projects have a lower probability of success (reaching goal amount). In case of failure, the pledged amounts are not transferred to the project creators (“all-or-nothing” mechanism). The coefficient estimates of the control variables also show that *Duration, Daily Activity,* and *Goal Amount* (*Log Goal*) negatively affect the success measures, while higher *Waiting Time* and being *Featured* by Kickstarter have a positive effect.

**Fig. 1:**
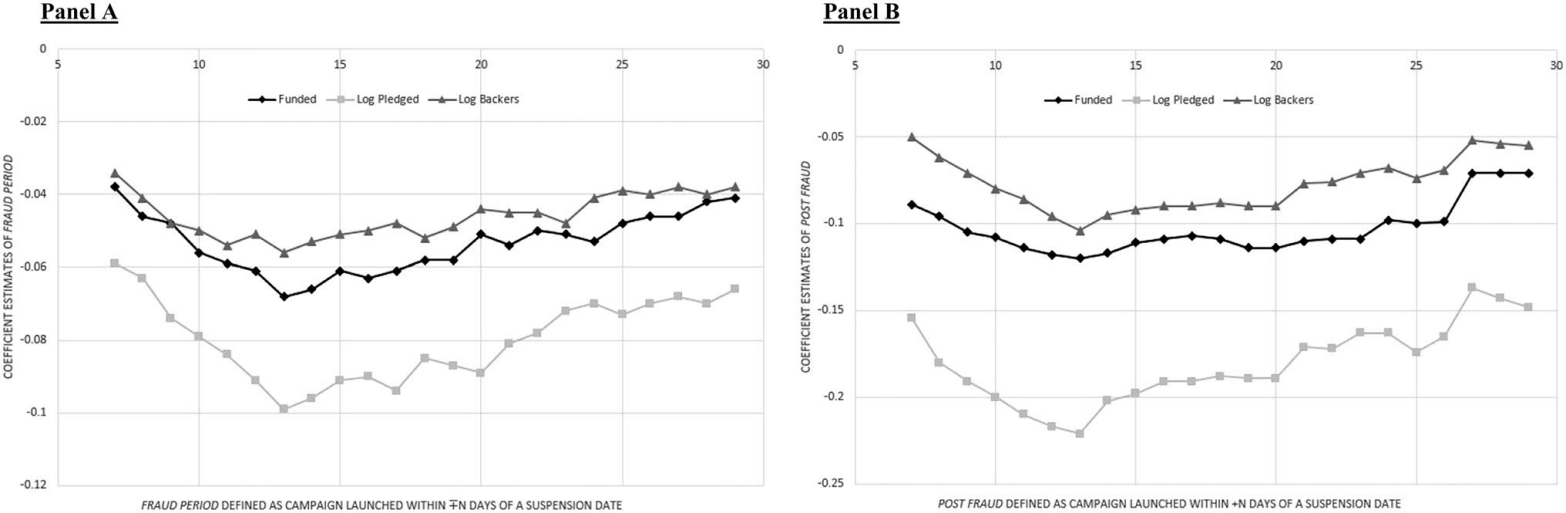
Sensitivity analysis. Panel **A** [Panel **B**] shows the estimated *Fraud Period* [*Post-Fraud*] dummy variable regression coefficients in Specifications (1)–(3) of Table 9, Panel A [Table 9, Panel B], using alternative classification schemes for campaigns affected by suspension announcements (*Fraud Period* and *Post-Fraud*) for the success measures as dependent variables (*Funded* (logit), *Log Pledged* (OLS), and *Log Backers* (OLS)). N (ranging from 7 to 29 days) determines the number of $\mp$ [$+$] days considered in the *Fraud Period* [*Post-Fraud*] dummy variable definition. All calculated coefficients are statistically significant at least at the 5% level

Next, we examine the sensitivity of our results to changes in the definition of the *Fraud Period* [*Post-Fraud*] dummy (in the baseline, we consider 14 days around [*Pre/Post*] the suspension date). We extend the period day-wise to twenty-nine. We then reduce it to 7 days around the suspension date, and repeat the regressions from Table 9, plotting the coefficient for the variable of interest, *Fraud Period* [*Post-Fraud*] in Fig. 1, Panel A [Panel B]*.* We expect to find the most negative coefficients when the platform-wide effects are most severe, i.e., when our sample of affected campaigns are in their first or last weeks of collecting funds. Shortening or extending the observation period from the 14-day definition should result in higher coefficient estimates (i.e., lower absolute values of the *Fraud Period* and *Post-Fraud* negative coefficients). This is because an overly short period does not capture the effect in full, while an overly long period dilutes the platform-wide effect. This results graphically in a V-shaped pattern.

From Fig. 1, Panel A [Panel B], and in line with our reasoning, we observe the strongest effect for the 13 days around the suspension date [13 days pre- and post-suspension announcement]. This fades slowly when we increase or decrease the number of days. The observed form reconciles with the V-shaped pattern. We interpret this as further support for the platform-wide negative consequences after the suspension of campaigns that slipped through Kickstarter’s initial screening, received a certain level of funding, and were canceled last minute.

We find strong evidence for Hypothesis 4, that large public suspensions by Kickstarter (as identified by our filter criteria described above) have noticeably damaging effects on other funding activities. This can potentially hamper entrepreneurship, and negatively affect the economy, employment, and innovation. It also raises interesting policy implications, namely, that platforms’ efforts to mitigate fraud should be focused more strongly on pre-screening mechanisms than on later project suspensions.^23^

## Conclusion

This paper is the first to provide an in-depth examination of the factors associated with a higher probability of fraudulent behavior in crowdfunding, and to analyze the short-term consequences of breaches of trust in the market. We provide evidence that legal enforcement by third parties such as the Federal Trade Commission or regional courts is rare. Because the penalties are usually small, focus should be on the pre-screening procedures and the liability of crowdfunding platforms.

We contribute to the literature by providing a practical (albeit not legal) definition of fraud in the crowdfunding market, and by identifying a comprehensive sample of campaigns associated with fraudulent behavior. We document campaign- and creator-related factors that tend to differ between fraudulent campaigns and a sample of non-fraudulent matched campaigns. We posit that these factors could be used by platforms to develop fraud-predicting models and fraud-preventing methods. We also provide the first empirical evidence of the effect of possible breaches of trust in the market on crowdfunding success. We discuss the implications of our findings further next.

For crowdfunding platforms, our evidence shows that not all scams are detected *ex ante*. The lack of fraud detection might justify regulations requiring platforms to improve their pre-screening procedures. However, screenings can become obsolete as fraudsters adapt and learn new ways to avoid detection. Therefore, and as an alternative way to increase trust in the market, platforms could design mechanisms to hold project creators accountable after successful funding. For example, they could halt campaigns once funding goals are reached and service any unmet demand in the after-market. They could also retain any funds raised in excess of the goal as insurance for backers (see Belavina et al. (2020) for a theoretical discussion of these two options).

For policymakers, we believe regulators are correct in attempting to protect less sophisticated crowd members. Until recently, most crowdfunding laws targeted specific branches—primarily equity crowdfunding. Reward-based crowdfunding was less regulated except in a few jurisdictions, such as Germany (Klöhn et al., 2016). Regulators could require reward-based crowdfunding platforms to implement pre-screening for particular quality requirements, or prohibit large overcontributions. However, since contribution amount is usually tied to platform fees, regulatory intervention may be more helpful. Once dynamically adapting fraud detection models are implemented and mechanisms exist to hold campaign creators accountable, it should become safer to discuss the phenomenon of crowdfunding with old-fashioned securities lawyers *without* the need for a defibrillator!

For campaign creators, we emphasize the importance of signals of first-party enforcement, as well as project quality, in ensuring backers’ trust and successful funding. We show that incidences of fraud in the market can be damaging to campaigns. Creators can mitigate this risk by reducing information asymmetries and providing difficult to mimic signals of project quality. For crowdfunding backers, the factors we identify can help evaluate project riskiness in terms of the probability of observing misconduct.

Our empirical analysis has some clear limitations. First, not all crowdfunding fraud is detectable. Thus, we may underestimate the true probability of fraud, a challenge for any prediction model. However, we believe that, at least on Kickstarter, it is unlikely that large-scale fraudulent campaigns go undetected. Small-scale fraud should be examined independently, given that its dynamics most likely differ from what we investigate here.

Second, and more importantly, we cannot legally prove the existence of any outright fraud campaigns. Our context does not allow us to empirically test whether creators have misappropriated funds, or developed low-quality products because of poor effort. We also cannot determine whether a judge would consider the “fraudulent” creators in our sample as simply incompetent. As a result, we use the words “fraud,” “misconduct,” and “fraudulent behavior” interchangeably throughout this study. We have tried to be as strict as possible about defining our criteria for including a campaign in the fraud sample.

Our study opens avenues for future research on fraud detection models for reward-based crowdfunding, as well as other forms (e.g., equity crowdfunding). In unreported tests, we examined whether concurrent projects in the same category where fraud occurred experienced more severe consequences. Our results revealed no evidence of statistically significant differences across categories. This may suggest that the borders between categories are somewhat “blurred” in a crowdfunding context (compared to, e.g., publicly listed firms). Also, backers do not seem to differentiate between categories in response to visible suspensions. However, future research could explore backers’ reactions to fraud (or other shocks).

We posit that, once equity crowdfunding emerges more fully in the U.S., we will observe different twists in fraud. This is because the campaigns are more complex, involve higher investment amounts, and usually comprise an entire venture. We expect the nature of fraud to adapt as well, and to require more sophisticated detection mechanisms. Note that, under a reward-based model, fraud generally occurs because founders do not develop the promised products or misuse funds. Under equity crowdfunding, founders may engage in a whole realm of unethical or illegal activities, such as running several start-ups at a time, violating fiduciary duties, or engaging in asset substitution and risk shifting. These can be more challenging to detect. But we believe our predictions will offer interesting avenues for empirical research as the market develops.

### Electronic supplementary material

Below is the link to the electronic supplementary material.

Supplementary file1 (DOCX 69 kb)

## Appendix

See Table 10.

**Table 10:** Correlation matrix

		1	2	3	4	5	6	7	8	9	10	11	12	13	14	15	16	17	18	19
1	*Creator-backed projects*	1																		
2	*Creator-created projects*	0.38*	1																	
3	Waiting time (months)	0.35*	0.35*	1																
4	Formal name	0.12*	0.04	0.14*	1															
5	Natural person	− 0.11*	− 0.03	− 0.14*	− 0.81*	1														
6	*# External links*	0.13*	0.07	0.12*	0	0.01	1													
7	*Facebook*	0.08	0.05	0.07	0.15*	− 0.17*	0.26*	1												
8	Facebook_Page	− 0.01	0	− 0.01	− 0.09	0.09	0.42*	0.49*	1											
9	Facebook_Personal	0.09	0.06	0.11*	0.23*	− 0.3*	0.11*	0.81*	0.1*	1										
10	LinkedIn	− 0.03	− 0.04	− 0.01	0.18*	− 0.14*	0.23*	0.03	0	0.04*	1									
11	Log (FB connections)	0.11	− 0.01	0.07	− 0.07	0.01	0.36*	0.16*	0.44*	− 0.14	0	1								
12	*Duration*	0.13*	− 0.09	− 0.08	0.01	− 0.09	− 0.01	0.08	0.06	0.07	0.06	0.07	1							
13	*Min. pledge amount*	− 0.05	0.07	− 0.02	− 0.1*	0.1	− 0.09	0	0.04	− 0.02	0.01	0.01	− 0.01	1						
14	*No. of pledge categories*	0.07	− 0.04	0.05	0.03	0	0.11*	0.16*	0.12*	0.06	0.06	− 0.03	0.03	− 0.23*	1					
15	*ARI*	− 0.01	− 0.04	0.03	− 0.15*	0.11*	0.12*	0.04	0.04	0	− 0.06	0.04	0.1*	0.07*	− 0.02	1				
16	CL	− 0.03	− 0.1	0	− 0.18*	0.18*	0.1	0.06	0.04	0.02	0.02	0.04	0.1*	0.09*	− 0.06	0.8*	1			
17	FKG	− 0.05	− 0.07	0.01	− 0.15*	0.12*	0.1*	0.04	0.04	− 0.01	− 0.08	− 0.01	0.08	0.08*	− 0.05	0.95*	0.72*	1		
18	GF	0.01	0.06	0.08	− 0.05	− 0.01	0.09	0.03	0.03	− 0.02	− 0.14*	0.02	0.03	0.03	0.05	0.69*	0.15*	0.72*	1	
19	*Video pitch*	0.07	− 0.03	0.06	− 0.07	0.1*	0.1	0.12*	0.13*	0.08	0.05	0.14*	0.09*	0.02	0.04	0.1*	0.15*	0.11*	− 0.01	1

This table shows the Pearson correlation coefficients for all variables considered in the “Fraud Determinants Analysis.” Variables are either used as main variables in Control 1, Control 2, and Control 3 (in italic), or as alternative proxies in robustness checks (see Table 1, Panel B, for variable descriptions and calculation methods)

*Statistical significance at least at a 5% level

## Footnotes

1 See http://money.cnn.com/2013/06/17/technology/kickstarter-scam-kobe-jerky

2 See http://business.financialpost.com/fp-comment/extraordinary-popular-delusions-and-the-madness-of-crowdfunding

3 In Part A of Online Appendix, we provide a discussion on legal sanctions in crowdfunding market.

4 See http://www.crowdfundinsider.com/2014/03/34255-crowdfunding-fraud-big-threat.

5 See Kickstarter’s guidelines for a definition of credible communications in the case of a failed project: https://www.kickstarter.com/fulfillment.

6 Our observation period for identifying suspected fraudulent campaigns spans 2010 through 2015, and we classified the campaigns in April 2016. We re-checked all suspected fraud campaigns on December 31, 2018, and excluded those where rewards had ultimately been delivered, a reason for late delivery or failure was provided, or backers were at least partially refunded.

7 This resulted in a further four exclusions from our base media reports fraud sample.

8 See http://www.theverge.com/2013/11/8/5081806/kickstarter-alleged-chargeback-fraud-hits-over-100-campaigns.

9 We use 2010–2015 as the sample period in order to ensure sufficient time (until 2018) to identify “suspected fraud” campaigns, especially in cases where rewards were not delivered.

10 In unreported tests, we examined the differences in means across all independent variables used in “determinant of fraud” analyses between fraudulent campaigns identified via *Kickscammed* vs. those identified via *News Search*. The results revealed no substantial differences in means.

11 The chronological sequence of the initiation date, campaign categories, and raised volumes in USD of fraudulent campaigns are in Panel C of Table 2. Fraud campaigns are most common in the “Technology” category (56 cases), where they have also raised the largest amounts (more than $11 million). Fraud campaigns by country for each respective year are shown in Panel D. In our sample, fraud cases occurred most frequently in the U.S. (171 cases); the U.K. (8 cases); and Canada (7 cases); followed by Israel (2); and Australia, China, Germany, Hong Kong, and Spain (1 each).

12 In an unreported table, we checked the quality of the PSM algorithm for our main analysis by using logit estimates for the probability of a campaign being fraudulent. We find that all variables (*Goal Amount*, *Country*, *Year*, and *Category*) included in the PSM are well balanced between fraud and non-fraud campaigns, and thus there are no statistically significant differences between them. Consequently, our results are not driven by any differences in these variables.

13 See https://help.kickstarter.com/hc/en-us/articles/115005139813-Why-would-a-project-be-suspended.

14 In order to ensure sufficient time for the potential suspension to have affected campaigns posted around the same time, we set this date as three months before our last funded/failed campaign has ended (i.e., December 31, 2018). Note that maximum campaign length is ninety days.

15 In order to show amounts in local currencies, Kickstarter converts non-USD currencies to USD using a static USD rate.

16 Note further that the thresholds we use for the four applied criteria did not have a strong effect on the fourteen identified cases. Relaxing the thresholds within certain margins would still result in the same fourteen suspended campaigns. For example, changing the first criterion to “at least 50% of campaign duration has passed” and the second to “campaign was suspended within 2 weeks of its scheduled deadline,” while retaining the same visibility criteria, results in the same fourteen cases.

17 We exclude “canceled” or “suspended” projects from our main sample because their success/failure do not depend on backers’ decisions.

18 For example, if Kickstarter suspends a campaign on March 15, 2015, the “Fraud Period” dummy equals 1 for all campaigns (either funded or failed) launched between March 1, 2015, and March 29, 2015. Our logic remains the same for any overlap between two suspension dates. For example, if suspension 1 is on March 15, 2015, and suspension 2 is on March 25, 2015, the “Fraud Period” equals 1 for all campaigns launched between March 1, 2015, and April 8, 2015.

19 See: https://www.kickstarter.com/help/faq/creator+questions.

20 All project creators on Kickstarter are required to provide official identification documentation. Each project is attributed to at least one natural person, and the name is publicly available on the campaign web page. The creator’s profile name can be the formal name or a fantasy name, but their information (first and family name) is readily available by clicking on the profile.

21 Panel A includes main category, year, month of year (January–December), day of month (first day to last day of respective month), and day of week (Monday–Sunday) fixed effects. Moreover, in Panel A, we control for a proxy for platform activity by calculating the average number of daily “live” campaigns during a project’s lifetime (*Daily Activity*). Panel B includes main category and year fixed effects. The time fixed effects are based on campaign launch dates.

22 Note that the average pledged amount/number of backers reported in Table 2, Panel C, is the average of the log-transformed variables.

23 In Part B of Online Appendix, we provide further robustness checks for our results.
