# Supplementary material for: Disentangling Crowdfunding from Fraudfunding
Source: J Bus Ethics. 2021 Oct 20;182(4):1103–28. doi: 10.1007/s10551-021-04942-w (PMC9905198; doi:10.1007/s10551-021-04942-w)
Supplement: Supplementary file 1 — Supplementary file1 (DOCX 69 kb) [file 10551_2021_4942_MOESM1_ESM.docx]

**Disentangling Crowdfunding from Fraudfunding**

Douglas Cumming^[[1]](#footnote-1)^, Lars Hornuf^[[2]](#footnote-2)^, Moein Karami^[[3]](#footnote-3)^, Denis Schweizer^[[4]](#footnote-4)^

**Online Appendix**

**Part A:**

**Legal Sanctions on Fraud in Crowdfunding Markets**

Law enforcement through third parties—the third leg of the Trust Triangle—is an essential element to deter fraud in markets (Ehrlich 1973). Securities laws in the U.S. have several antifraud provisions that allow investors and the SEC to bring legal actions and enforce legal rules. These provisions apply in the context of a purchase or sale of a security. While equity crowdfunding and peer-to-peer lending issuers almost inevitably offer securities (Bradford 2012), neither donation- nor reward-based crowdfunding includes securities as defined under the Securities Act § 2(a)(1) or the Exchange Act § 3(a)(10). Thus, backers cannot recover damages from fraudulent campaign creators under U.S. securities laws. Moreover, the SEC has no jurisdiction over these matters, and, consequently, cannot impose fines or achieve injunctive relief, as would be possible for fraudulent security offerings on traditional capital markets.

However, many jurisdictions provide common law or general civil law code fraud actions, even if no securities are involved. In the U.S., for example, backers can take action under state law if the following five elements are present: 1) the creator makes a false statement related to a material fact, 2) the creator knows that the statement is untrue, 3) the creator intends to deceive the backer, 4) the backer reasonably relied on the statements of the creator when making a decision to invest, and 5) the backer was injured, which, in a crowdfunding context, is likely if funds are lost and no product was delivered. In order to recover money pledged by crowdfunding, a backer would, therefore, have to show a court that the campaign creator committed a fraud and that the backer relied on false statements in choosing to invest.

One problem with private remedies is that the amount of the claims often does not justify the costs of litigation. Class actions may be potentially suitable in cases where many backers deceived by the same creator can consolidate their claims. Given that the pledges of most crowdfunding contributions are extremely small, even class actions may not be feasible because legal cases are too expensive, time-consuming, and emotionally exhausting relative to the expected refund. Thus, the most effective remedies need to come through government agencies.

Finally, there are criminal provisions prohibiting fraud in a crowdfunding context. The Federal Trade Commission (FTC) has jurisdiction when crowdfunding involves the sale of a good (which is typically true with pre-purchases and, potentially, in cases when rewards are offered). Importantly, the FTC has the authority to impose monetary penalties on fraudulent campaign creators. It may also obtain civil penalties if fraudulent entrepreneurs persistently violate its standards.

Currently, we are aware of only a single case where the FTC acted on a crowdfunding fraud: a case involving a campaign set up by Erik Chevalier, which was known as *The Doom That Came To Atlantic City!* and was created under the business synonym *The Forking Path, Co.* In June 2012, 1,246 backers had pledged a total of USD $122,874 for Chevalier to develop a new board game. As part of the campaign, he promised backers that they could pre-purchase a copy of the game as well as specially designed action figures. However, after fourteen months, Chevalier declared that he had terminated the project and intended to refund the backers. According to the FTC, instead of creating the game, Chevalier had spent most of the money on his own expenses, such as rent, a move to Oregon, personal equipment, and licenses for an unrelated project^[[5]](#footnote-5)^. As a result, the FTC filed a complaint for a permanent injunction, followed by an order of judgment for USD $111,793.71 (*FTC v. Chevalier*, No. 3:15-cv-01029-AC [D. Or. June 10, 2015]). The judgment was suspended, however, due to Chevalier’s inability to pay.

In another Kickstarter campaign called *Asylum Playing Cards*, Edward J. Polchlopek III, the president of Altius Management, LLC, attracted 810 backers pledging a total of USD $25,146 in October 2012. In this case, the campaign creator promised backers he would print and market a deck of playing cards created by a Serbian artist. After failing to deliver the promised rewards and ending communication with the crowd in July 2013, the King County Superior Court ordered a total of USD $668 in restitution be made to thirty-one backers living in Washington State. Furthermore, court commissioner Henry Judson ordered another USD $1,000 per violation (USD $31,000 in total) in civil penalties for violating the state Consumer Protection Act, as well as USD $23,183 to cover the costs and fees of bringing the case (*State of Washington v. Polchlopek*, No. 14-2-12425-SEA [Wash. Super. Ct. April 30, 2014]).

The inactivity of the FTC and the lack of private legal actions does not necessarily imply that fraudulent behavior is absent in crowdfunding markets. The FTC’s inactivity can be partially attributed to the high costs of verifying contracts (Lacker and Weinberg 1989; Townsend 1979) in crowdfunding context. This is because, in many cases, it is extremely difficult and costly to prove that the creator intended to deceive backers. As a result, many backers and government agencies may be disincentivized from bringing presumably fraudulent cases in front of a court. Consequently, it is also important to investigate suspicious crowdfunding activities in order to methodically construct a comprehensive and reliable sample of fraudulent campaigns.

To summarize, fraudsters in a reward-based crowdfunding campaign may anticipate being detected as the campaign progresses and the delivery date approaches. Despite the weak incentives of backers, who may have pledged only small amounts, to bring legal action, fraudsters are still subject to prosecution by the FTC or by state attorneys general. However, the inactivity of government agencies, such as the FTC, until 2019 has signaled the overall absence of the third leg of the Trust Triangle—legal liability. Thus, the lack of private and government actions may provide fraudsters with sufficient incentives to engage in deceptive activities.

**Part B:**

**A Robustness Check**

In this section, we check the robustness of our findings by examining the impact of identified signals of first-party enforcement on the success of crowdfunding campaigns in the 2010 to 2018 Kickstarter sample (controlling for main determinants of success). We aim to show the relevance of these signals in predicting success, especially when there exists a perceived signal of weak related-party enforcement in the market.

We find that the identified signals of strong first-party enforcement positively affect campaign success, which can be thought of as backers’ trust level in terms of contributed amounts, and this effect is larger when a “late” platform enforcement (perceived by backers) occurs. We highlight the importance of related-party enforcement and platform scrutiny before projects are posted, especially since crowdfunding platforms do not have mechanisms in place for enforcing accountability (e.g., by charging an insurance fee proportional to the overcontribution to the campaign) once the funds are transferred to the campaign creator.

In Table A1, we aim to relate the two parts of the empirical analyses, and examine the effect of identified signals of first-party enforcement (associated with lower probability of fraud) on success. In this table, we analyze the determinants of Success measured by Funded (logistic regression; coefficients are the logs of the odds ratios), Log Pledged (OLS regressions), and Log Backers (OLS regressions) in fraud vs. non-fraud period and examining the effect of main variables identified initially as determinants of fraud. In panel A, we include only the campaigns being affected by suspension announcements (Fraud Period =1) with goal amounts of at least $100 that were posted after January 1, 2010, and ended before December 31, 2018 (to a total of 41,229 affected campaigns). In panel B, we include only the campaigns not being affected by suspension announcements based on our definition (Fraud Period =0) to a total of 230,742 campaigns.

In summary, Table A1 provides further evidence for robustness of our results as we found that factors that are negatively (positively) associated with probability of observing fraudulent behavior, positively (negatively) predict campaign success, in both panels. Moreover, we show that coefficients are, in vast majority of cases (except the coefficient on *Facebook*), larger for the affected sample (Panel A) compared to the not affected sample (Panel B), possibly suggesting that signals of first-party enforcement play a more important role in determining backers’ trust level, when there exists a signal of weak related-party enforcement.

*—Please insert Table A1 about here—*

**References**

Bradford, S. (2012). Crowdfunding and the federal securities laws. *Columbia Business Law Review,* *1*, 1–150.

Ehrlich, I. (1973). Participation in illegitimate activities: A theoretical and empirical investigation. *Journal of Political Economy,* *81*(3), 521–565.

Lacker, J. M., & Weinberg, J. A. (1989). Optimal contracts under costly state falsification. *Journal of Political Economy,* *97*(6), 1345–1363.

Townsend, R. (1979). Optimal contracts and competitive markets with costly state verification. *Journal of Economic Theory,* *21*(2), 265–293.

**Table A1: Multivariate Analysis of Platform-wide Consequences of Fraud (Robustness Check)**

In this table, we analyze the determinants of *Success* measured by *Funded* (logistic regression; coefficients are the logs of the odds ratios), *Log* *Pledged* (OLS regressions), and *Log Backers* (OLS regressions) in fraud (Panel A) vs. non-fraud (Panel B) period. All non-dummy variables are winsorized at the 1% level on both sides. Controls include *Waiting Time, Featured, Log Goal,* and *Daily Activity*. Robust standard errors are one-way-clustered by campaign category. *t*-statistics are in parentheses. ***, **, and * indicate statistical significance at the 1%, 5%, and 10% levels, respectively.

**Panel A**

|  | **(1)** | **(2)** | **(3)** |
| --- | --- | --- | --- |
|  | **Funded** | **Log Pledged** | **Log Backers** |
| # Creator-Backed Projects | 0.104^**^ | 0.026^*^ | 0.018^*^ |
|  | (2.35) | (2.01) | (2.38) |
| # Creator-Created Projects | 0.636^***^ | 0.320^***^ | 0.196^***^ |
|  | (6.14) | (6.98) | (5.89) |
| # External Links | 0.132^***^ | 0.085^***^ | 0.049^***^ |
|  | (6.25) | (3.42) | (2.97) |
| Facebook | 0.664^***^ | 1.206^***^ | 1.985^***^ |
|  | (8.33) | (4.44) | (4.98) |
| Duration | -0.032^***^ | -0.004^***^ | -0.003^***^ |
|  | (-4.19) | (-2.98) | (-3.96) |
| Min. Pledge Amount | 0.036 | 0.006^*^ | 0.004^*^ |
|  | (0.65) | (2.08) | (2.01) |
| No. of Pledge Categories | -0.270^***^ | -0.045^***^ | -0.030^***^ |
|  | (-3.46) | (-2.65) | (-3.24) |
| ARI | 0.328^**^ | 0.241^***^ | 0.150^***^ |
|  | (2.22) | (2.98) | (3.65) |
| Video Pitch | 0.225 | 0.082 | 0.030 |
|  | (0.65) | (0.48) | (0.85) |
| Constant | 6.079^***^ | 11.528^***^ | 6.547^***^ |
|  | (6.41) | (12.24) | (17.70) |
| Controls | Yes | Yes | Yes |
| Category FE | Yes | Yes | Yes |
| Year FE | Yes | Yes | Yes |
| Observations | 41,229 | 41,229 | 41,229 |
| Mean VIF | 1.23 | 1.23 | 1.23 |
| Maximum VIF | 1.84 | 1.84 | 1.84 |
| Adjusted *R*^2^ |  | 0.222 | 0.268 |
| Pseudo *R*^2^ | 0.190 |  |  |

**Table A1: Multivariate Analysis of Platform-wide Consequences of Fraud (Robustness Check)—*continued***

**Panel B**

|  | **(1)** | **(2)** | **(3)** |
| --- | --- | --- | --- |
|  | **Funded** | **Log Pledged** | **Log Backers** |
| # Creator-Backed Projects | 0.102^*^ | 0.021 | 0.015 |
|  | (2.01) | (1.46) | (1.70) |
| # Creator-Created Projects | 0.503^***^ | 0.303^***^ | 0.184^***^ |
|  | (7.47) | (9.54) | (7.32) |
| # External Links | 0.111^***^ | 0.084^***^ | 0.046^***^ |
|  | (7.32) | (5.42) | (4.84) |
| Facebook | 0.806^***^ | 1.065^***^ | 1.909^***^ |
|  | (8.75) | (5.19) | (5.65) |
| Duration | -0.027^***^ | -0.002^***^ | -0.001^***^ |
|  | (-6.21) | (-4.88) | (-4.96) |
| Min. Pledge Amount | 0.035 | 0.010 | 0.006 |
|  | (0.87) | (1.24) | (1.51) |
| No. of Pledge Categories | -0.257^***^ | -0.044^***^ | -0.030^***^ |
|  | (-4.36) | (-3.65) | (-3.94) |
| ARI | 0.304^*^ | 0.218^**^ | 0.137^**^ |
|  | (2.02) | (2.34) | (2.45) |
| Video Pitch | 0.165 | 0.071 | 0.062 |
|  | (0.95) | (1.45) | (1.32) |
| Constant | 3.056^***^ | 8.431^***^ | 4.449^***^ |
|  | (5.75) | (9.24) | (10.63) |
| Controls | Yes | Yes | Yes |
| Category FE | Yes | Yes | Yes |
| Year FE | Yes | Yes | Yes |
| Observations | 230,742 | 230,742 | 230,742 |
| Mean VIF | 1.18 | 1.18 | 1.18 |
| Maximum VIF | 1.73 | 1.73 | 1.73 |
| Adjusted *R*^2^ |  | 0.219 | 0.248 |
| Pseudo *R*^2^ | 0.188 |  |  |

1. Douglas Cumming

   [cummingd@fau.edu](mailto:cummingd@fau.edu)

   Lars Hornuf

   [hornuf@uni-bremen.de](mailto:hornuf@uni-bremen.de)

   Moein Karami

   [moein.karami@concordia.ca](mailto:moein.karami@concordia.ca)

   Denis Schweizer

   [denis.schweizer@concordia.ca](mailto:denis.schweizer@concordia.ca)

   College of Business, Florida Atlantic University, 777 Glades Road, Boca Raton, Florida 33431, US [↑](#footnote-ref-1)
2. Faculty of Business Studies and Economics, University of Bremen, Max-von-Laue-Str. 1, 28334 Bremen, Germany [↑](#footnote-ref-2)
3. John Molson School of Business, Concordia University, 1450 Rue Guy, Montreal, Quebec H3H 0A1, Canada [↑](#footnote-ref-3)
4. John Molson School of Business, Concordia University, 1450 Rue Guy, Montreal, Quebec H3H 0A1, Canada [↑](#footnote-ref-4)
5. See: https://www.ftc.gov/news-events/press-releases/2015/06/crowdfunding-project-creator-settles-ftc-charges-deception [↑](#footnote-ref-5)
